# Gut germinal center regeneration and enhanced antiviral immunity by mesenchymal stem/stromal cells in SIV infection

**DOI:** 10.1172/jci.insight.149033

**Published:** 2021-06-22

**Authors:** Mariana G. Weber, Chara J. Walters-Laird, Amir Kol, Clarissa Santos Rocha, Lauren A. Hirao, Abigail Mende, Bipin Balan, Juan Arredondo, Sonny R. Elizaldi, Smita S. Iyer, Alice F. Tarantal, Satya Dandekar

**Affiliations:** 1 Department of Medical Microbiology and Immunology and; 2 Department of Pathology, Microbiology and Immunology, University of California Davis, Davis, California, USA.; 3 Dipartimento di Scienze Agrarie Alimentari Forestali, Università di Palermo, Viale delle Scienze, Palermo, Italy.; 4 Center for Immunology and Infectious Diseases,; 5 California National Primate Research Center, and; 6 Departments of Pediatrics and Cell Biology and Human Anatomy, University of California Davis, Davis, California, USA.

**Keywords:** AIDS/HIV, Cellular immune response

## Abstract

Although antiretroviral therapy suppresses HIV replication, it does not eliminate viral reservoirs or restore damaged lymphoid tissue, posing obstacles to HIV eradication. Using the SIV model of AIDS, we investigated the effect of mesenchymal stem/stromal cell (MSC) infusions on gut mucosal recovery, antiviral immunity, and viral suppression and determined associated molecular/metabolic signatures. MSC administration to SIV-infected macaques resulted in viral reduction and heightened virus-specific responses. Marked clearance of SIV-positive cells from gut mucosal effector sites was correlated with robust regeneration of germinal centers, restoration of follicular B cells and T follicular helper (Tfh) cells, and enhanced antigen presentation by viral trapping within the follicular DC network. Gut transcriptomic analyses showed increased antiviral response mediated by pathways of type I/II IFN signaling, viral restriction factors, innate immunity, and B cell proliferation and provided the molecular signature underlying enhanced host immunity. Metabolic analysis revealed strong correlations between B and Tfh cell activation, anti-SIV antibodies, and IL-7 expression with enriched retinol metabolism, which facilitates gut homing of antigen-activated lymphocytes. We identified potentially new MSC functions in modulating antiviral immunity for enhanced viral clearance predominantly through type I/II IFN signaling and B cell signature, providing a road map for multipronged HIV eradication strategies.

## Graphical abstract

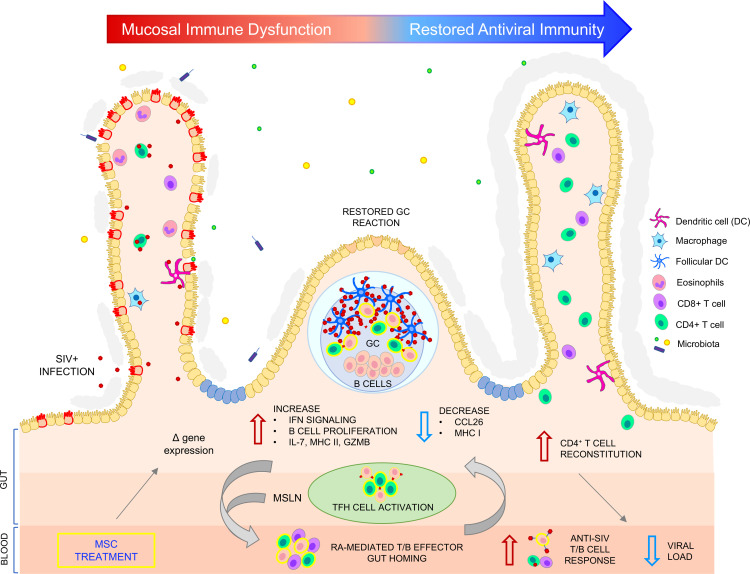

## Introduction

HIV disease progression is characterized by CD4^+^ T cell loss, chronic immune activation, and immune dysfunction (1). Although antiretroviral therapy (ART) is effective in suppressing HIV replication and reducing morbidity and mortality, it fails to eradicate latent HIV reservoirs and is unable to directly restore immune functions or repair damaged lymphoid tissues (2, 3). Novel approaches to enhance host immunity for targeted viral eradication and complete immune recovery are warranted.

The gut-associated lymphoid tissue (GALT) is an early site for viral replication and establishment of stable viral reservoirs. Early pathogenic effects of HIV infection include severe depletion of gut mucosal CD4^+^ T cells and epithelial barrier disruption (4, 5). Induction of lymphoid follicle damage and germinal center (GC) loss also occur early in the GALT and persist through chronic viral infection, leading to ineffective T follicular helper (Tfh) and B cell interactions (6). These changes contribute to impaired mucosal immunity and incomplete viral clearance (7). Effective viral suppression has been associated with preservation of lymphoid follicle structure and T and B cell functionality in HIV-infected elite controllers and in SIV-infected nonhuman primates (8, 9). GC integrity is important for supporting better antigen presentation to generate effective antibody responses to vaccines (10). A vaccine-induced B cell gene signature was associated with protection against HIV and SIV infections, highlighting its role in viral control (11). New strategies to enhance gut mucosal immunity through GC renewal and suppression of mucosal inflammation have great potential to boost immune control of viral infection.

Mesenchymal stem/stromal cells (MSCs) are somatic multipotent stromal cells with antiinflammatory and immunomodulatory properties (12). The safety profile of allogeneic bone marrow–derived MSC treatment has been demonstrated in several nonhuman primate studies and human clinical trials (13). Specifically, multiple infusions of allogeneic MSCs in HIV-infected patients had no adverse effects on viral loads or CD4^+^ T cell numbers (14). MSCs infused via i.v. home to the gut mucosa in animal models of intestinal inflammation and radiation-induced intestinal injury, including nonhuman primates (15, 16). MSCs also restored impaired gut mucosal immunity in aged mice and enhanced vaccine-specific antibody response (17). MSC therapy has been extensively investigated in inflammatory diseases and cellular injury models, but its effects on the GALT microenvironment and mucosal immunity in SIV/HIV infection have not yet been investigated. Our understanding of the MSC-mediated mechanisms of repair and regeneration of immunity is very limited. This necessitates in-depth investigations of in vivo early effects of MSCs in inflamed lymphoid microenvironments and interactions with immune cells.

In this study, we utilized allogeneic bone marrow–derived MSCs in the SIV-infected rhesus macaque model of AIDS to investigate whether MSC administration in vivo would reduce gut inflammation and restore mucosal lymphoid functions for the revival of antiviral immunity. Based on an integrated analysis of immunological, virological, transcriptomic, and metabolic data, we found that MSC treatment restored mucosal lymphoid follicle structure and boosted antiviral immunity, which correlated with viral suppression and immune recovery. Robust regeneration of GCs and restoration of follicular B and T cells were associated with enhanced antigen presentation in B cell follicles and gene expression enriched for type I/II interferon signaling, B cell proliferation and IL-7. These changes were reflected in the activation of metabolic pathways associated with enhanced immunity and viral reduction. Our findings highlight the potential of MSCs to subvert viral pathogenic effects on GALT structure and function, which could be leveraged for enhancing antiviral immunity and for developing synergistic HIV cure strategies.

## Results

### Reduction of plasma SIV loads and viral clearance from GALT effector sites in MSC-treated animals.

**Figure 1:**
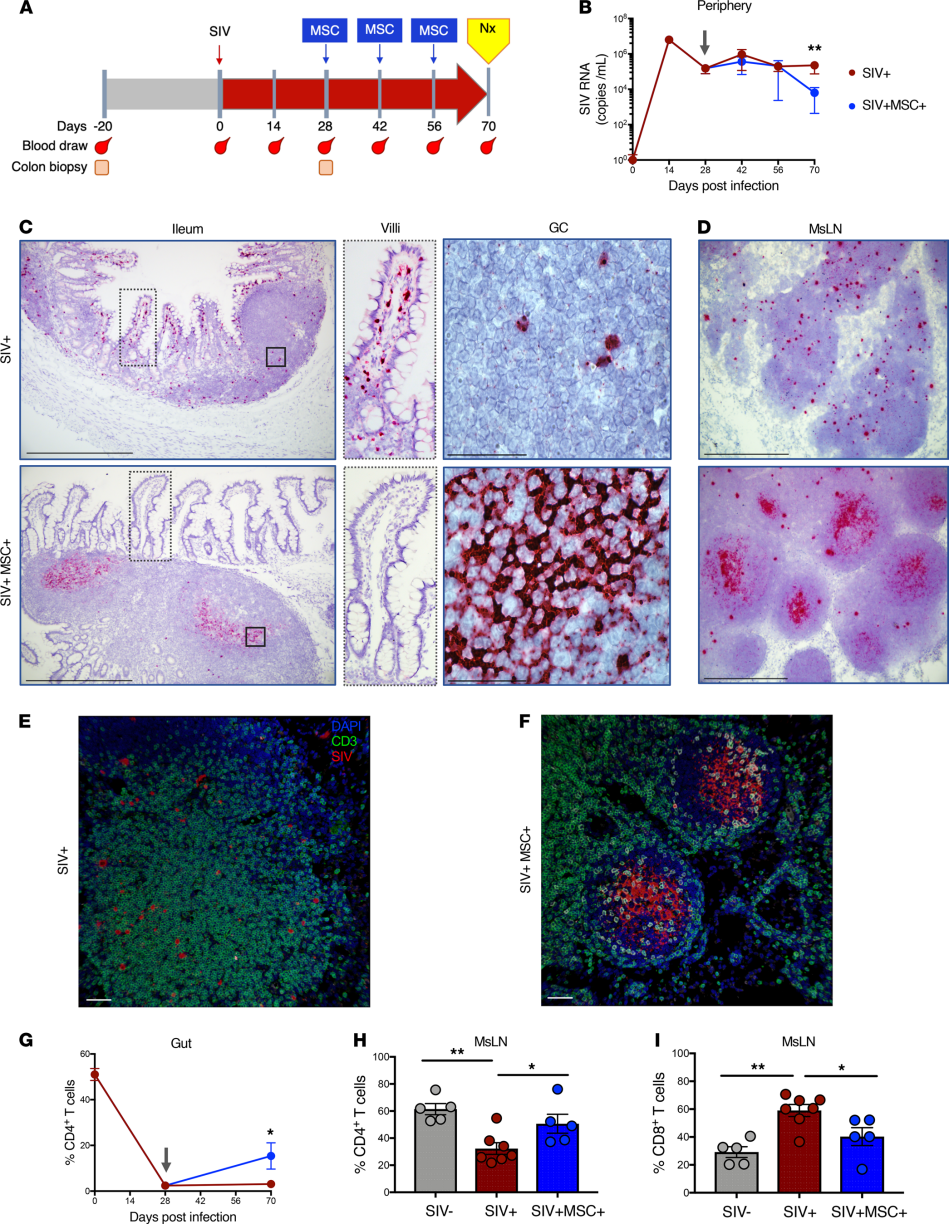
MSC promotes peripheral virus reduction with clearance from effector sites and restoration of CD4^+^ T cells in the gut lymphoid follicles. (**A**) Study design and sample collection. Nx indicates necropsy. (**B**) Viral RNA loads were measured in longitudinal plasma samples at 0, 14, 28, 42, 56, and 70 days after infection by qPCR (SIV^+^ *n* = 7, SIV^+^MSC^+^ *n* = 5). (**C** and **D**) Detection of SIV RNA in ileum and MsLNs by in situ RNA hybridization. Representative images show SIV RNA in villi and lymphoid follicle (LF) during chronic infection (RNAscope, 10× magnification). RNA visualized as a chromogenic magenta stain; 100× magnification of villi showing the presence of virus in SIV^+^ animals and its absence in SIV^+^MSC^+^, and GC in ileal tissue showing individual infected cells (SIV^+^) and viral trapping on follicular DC network (SIV^+^MSC^+^). Inspection of MsLNs demonstrated well-developed GCs in the SIV^+^MSC^+^ group with SIV viral RNA localizing preferentially within GCs compared with SIV^+^ animals. Magnification 10× and 100×. Scale bars: 250 μm and 25 μm. (**E** and **F**) Dual in situ RNA hybridization and IHC analysis of MsLN of SIV^+^ and SIV^+^MSC^+^, showing viral RNA (red) and T cells (green). Magnification 20×. Scale bars: 50 μm. (**G**) Shown are longitudinal percentages of CD4^+^ T cells in the gut cells after gating on lymphocytes, singlets, live cells, CD45^+^, and CD3^+^ T cells (SIV^+^ *n* = 7 and SIV^+^MSC^+^ *n* = 5). (**H** and **I**) Percentage of CD4^+^ and CD8^+^ T cells in MsLNs (SIV^–^ *n* = 5, SIV^+^ *n* = 7, and SIV^+^MSC^+^ *n* = 5) at 70 days after infection. Data represent the mean (± SEM) for each time point. Significance was determined using the Mann-Whitney *U* test (**B** and **G**) or 1-way ANOVA with Holm-Sidak post hoc testing for multiple comparisons (**H** and **I**). *P < 0.05 and **P ≤ 0.01. Gray arrow represents first MSC administration.

To investigate the effects of MSC on SIV infection and T cell subset distribution, we determined plasma viral RNA loads by quantitative PCR (qPCR) and detected SIV RNA–positive cells in the gut tissue compartment by RNAscope. Changes in the prevalence of CD4^+^ and CD8^+^ T cell subsets in PBMCs and lamina propria lymphocytes were detected by flow cytometric analysis. Rhesus macaques (*n* = 17) were i.v. infected with SIV, and 5 animals were treated with i.v. infusions of MSC at 28, 42, and 56 days after SIV infection; the remaining animals served as untreated SIV-infected controls (Figure 1A). Adverse effects of MSC infusion were evaluated, and no increased respiratory rate, anaphylaxis, or thrombosis were displayed. The rationale for initiating MSC administration at 28 days after infection was that animals had transitioned from the primary acute stage to the chronic phase of SIV infection. At this stage, stable viral reservoirs are established in lymphoid tissues and viral set points are reached in the peripheral blood. This is coincident with severe mucosal CD4^+^ T cell depletion, gut epithelial barrier disruption, and mucosal inflammation (18, 19). Animals were euthanized at 70 days after infection for a comprehensive investigation of early impact of MSCs on the reversal of viral pathogenic effects through evaluation of immunological and virological changes. Peripheral blood samples were collected prior to and after SIV infection. Colonic biopsies were collected prior to infection to provide baseline CD4^+^ T cell data, 28 days after infection to determine effects on mucosal CD4^+^ T cell depletion, and at 70 days after infection to evaluate effects of MSC treatment (Figure 1A). A significant decrease in plasma SIV RNA levels was noted in MSC-treated animals compared with untreated SIV-infected controls, indicating reduction of viral replication (Figure 1B). We previously reported that viral RNA-positive cells are widely distributed in the gut microenvironment but not evenly spread across the tissue (19). This is attributed to the diverse composition and compartmentalization of the cells within the gut. We utilized RNAscope in situ hybridization to detect and localize SIV RNA–positive cells in the gut tissue samples. We found marked differences in the levels and localization of SIV RNA in MSC-treated compared with untreated SIV-infected animals. SIV RNA–positive cells were widely distributed throughout the intestinal lamina propria but were mostly absent from lymphoid follicles in untreated animals (Figure 1C). This finding is in agreement with previous reports on viral dissemination during disease progression in HIV and SIV infections (19, 20). In a striking contrast, there was a marked reduction in the level of SIV RNA–positive cells in intestinal lamina propria of MSC-treated animals. Instead, SIV RNA was predominantly localized to lymphoid follicles and trapped in the follicular DC network as evidenced by the reticular pattern (Figure 1C). Similar changes were detected in mesenteric lymph nodes (MsLNs, Figure 1D). In confirmation with the RNAscope data, IHC analysis demonstrated that the SIV RNA was broadly disseminated in the extrafollicular zone of untreated animals (Figure 1E) but was restricted to the B cell follicle in treated animals (Figure 1F). Since it is well recognized that CD4^+^ Tfh cells support viral persistence (21), we sought to investigate whether viral RNA in the B cell follicle of MSC-treated animals was also associated with the Tfh cell reservoir. High-magnification analysis (100×) in MsLNs suggested that SIV RNA was not associated with Tfh (CD3^+^ PD-1^+^) cells but was localized within the follicular DC network (Supplemental Figure 1A; supplemental material available online with this article; https://doi.org/10.1172/jci.insight.149033DS1).

We hypothesized that viral clearance from the gut effector sites would lead to partial restoration of mucosal CD4^+^ T cells. Although no significant difference was detected in the frequency of CD8^+^ T cells (Supplemental Figure 1B), we found a statistically significant increase in mucosal CD4^+^ T cell percentage in isolated mononuclear cells from colon tissues of MSC-treated macaques (Figure 1G). In the MsLNs, the frequency of CD4^+^ T cells increased with a proportional decrease in CD8^+^ T cells from MSC-treated versus untreated animals (Figure 1, H and I). Thus, viral clearance from gut mucosal effector sites correlated with the partial restoration of mucosal CD4^+^ T cells.

### MSC-treated animals display a gut mucosal transcriptional signature enriched for type I/II IFN signaling, antigen presentation, and B cell proliferation.

**Figure 2:**
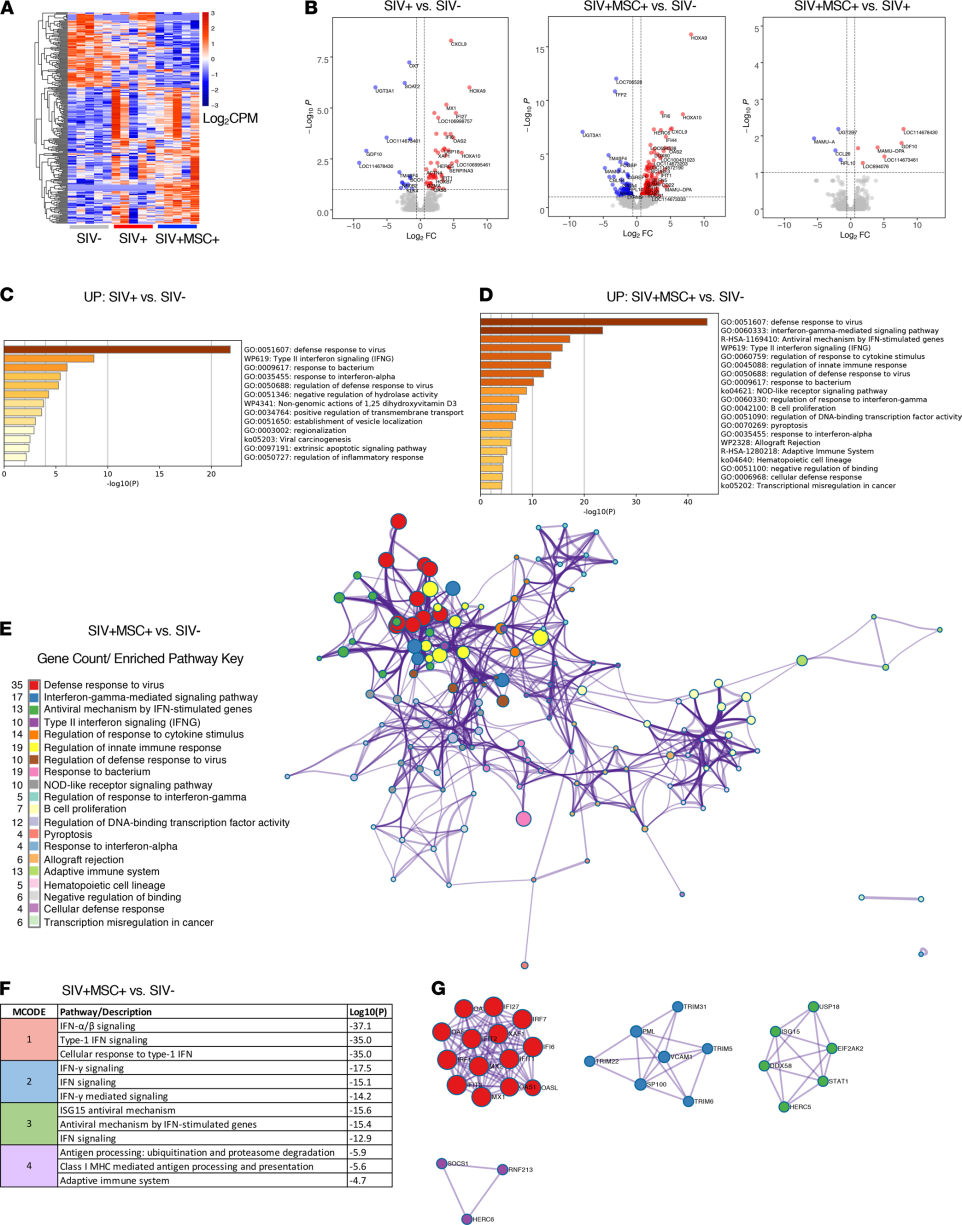
Transcriptomic signature of enhanced antiviral immunity in MSC-treated SIV-infected rhesus macaques. (**A**) Heatmap displays log-transformed counts per million (CPM) of all differentially expressed genes (DEGs) for each animal in control and treatment cohorts (*n* = 5/group). Significance defined as FDR < 0.1 and FC > ±1.5; a negative binomial distribution model of gene counts was used in Edge R. (**B**) Volcano plots show the log_2_ fold change (log_2_FC) versus log_–10_ (*P*) for all detected gene transcripts where *P* represents the Benjamini-Hochberg (BH) FDR. Dotted lines represent significance threshold cutoffs. (**C–G**) Analyses and figures were generated through Metascape.org. (**C** and **D**) Bar graphs show upregulated, enriched biological pathways detected where *P* represents BH-FDR. Significant upregulated DEGs were used as input. SIV^+^MSC^+^ animals and SIV^+^ control group were compared with SIV^–^ controls to evaluate the magnitude of the gut mucosal immune response. (**E**) Network analysis showing term enrichment (node size) and interactions (edges) between the top 20 detected biological pathways (color). Significance determined using accumulative hypergeometric distribution and BH-FDR correction. (**F**) Table displays enriched MCODE modules as determined by a protein-protein interaction analysis. (**G**) Expressed proteins with known physical interactions, associations, and biological regulation in enriched modules.

To determine the molecular basis of viral reduction in the gut lamina propria and mucosal CD4^+^ T cell recovery in MSC-treated rhesus macaques, we performed transcriptomic analysis of ileum tissue collected at 70 days after infection from SIV-infected animals with and without MSC treatment and compared them with SIV-negative controls (*n* = 5/group, Figure 2A). The mucosal gene expression data were analyzed at 3 levels: differential gene expression, biological pathway analysis, and protein-protein interactions. First, we identified differentially expressed genes (DEGs) among the groups. The SIV-infected untreated group was compared with SIV-negative controls to establish the expression changes driven by the viral infection (Figure 2, A and B). We identified a total of 69 DEGs: 52 upregulated and 17 downregulated. Next, to ensure that all the MSC-driven changes in the gut were captured, the MSC-treated group was compared with SIV-negative and SIV-infected controls. Surprisingly, we found 229 DEGs (156 upregulated and 73 downregulated) in MSC-treated animals when compared with SIV-negative controls. This revealed that MSC infusion in the context of SIV infection induced greater changes in ileum than the chronic viral infection alone. A direct comparison of MSC-treated with untreated animals also revealed a gene signature consisting of 10 DEGs, with 6 upregulated and 4 downregulated gene targets (Figure 2B). Increased expression of MAMU-DPA (MHC class II) and growth differentiation factor 10 and 4 DEGs with unknown function was identified, warranting future investigation. A significant decrease in the expression of chemokine ligand 26 (CCL26), MHC class I (MAMU-A), ribosomal protein L10 (RPL10), and UDP glucuronosyltransferase family 2 member B7 (UGT2B7) was detected.

Next, a comprehensive pathway enrichment analysis was performed with each set of DEGs from all 3 comparisons (SIV^+^MSC^+^ vs. SIV^+^, SIV^+^MSC^+^ vs. SIV^–^, and SIV^+^ vs. SIV^–^) using the online platform Metascape (22). As expected, we observed enriched mucosal antiviral immune pathways in SIV-infected animals (Figure 2C). However, MSC-treated gut tissue displayed more pronounced changes and a higher magnitude of difference for the gene expression of defense response to virus and IFN type I/II signaling. We further identified enrichment for genes associated with regulation of cytokine signaling and innate immunity, NOD-like receptor signaling, and B cell proliferation, reflecting the restoration of a robust and effective mucosal anti-SIV immune response (Figure 2, C and D). These pathways were not observed in untreated animals. These findings were in support of viral reduction in gut mucosal tissues as evidenced by RNAscope analysis (Figure 1, C and D). As previously reported, SIV infection was associated with impaired gut function, including nutrient digestion, which is related to epithelial disruption (18, 23) (Supplemental Figure 2, A and B). To understand the integration between significantly enriched pathways, we generated a network displaying gene clusters (nodes) and respective interactions (edges) (Figure 2E and Supplemental Figure 2C). It was apparent from the network that MSC-treated animals displayed a more coordinated and integrated antiviral innate and adaptive immune response in the gut. This was shown by the abundant interactions between the pathway gene clusters in MSC-treated animals compared with untreated controls.

Finally, we identified meaningful protein-protein interactions based on these gene networks. A total of 3 and 4 protein interaction modules were identified in untreated and MSC-treated animals (Figure 2, F and G, and Supplemental Figure 2, D and E). These groups shared enrichment of type I IFN signaling and antiviral mechanism modules. The MSC-treated animals could be distinguished from untreated controls based on the expression and interplay of IRF1, IRF7, and OASL as well as STAT1 and DDX58 proteins, which are identified within the type-1 IFN module (MCODE-1) and antiviral mechanism module (MCODE-3). Enriched IFN-γ–stimulated interactions (MCODE-2) and antigen presentation (MCODE-4) pathways were also detected. Overall, pronounced antiviral mucosal transcriptomic changes paralleled the viral clearance from the ileal lamina propria and colonic CD4^+^ T cell recovery observed in MSC-treated animals (Figure 1). In addition, alteration of MHC class I/II molecules in treated animals compared with both controls suggests a strong role for MSC modulation of antigen presentation pathways. This gene expression signature reveals an exciting potential of MSCs to restore robust and effective antiviral mucosal immunity during chronic SIV infection and will guide future investigations to determine underlying mechanisms.

### MSC treatment induces restoration of gut GCs and Tfh and B cell subsets.

**Figure 3:**
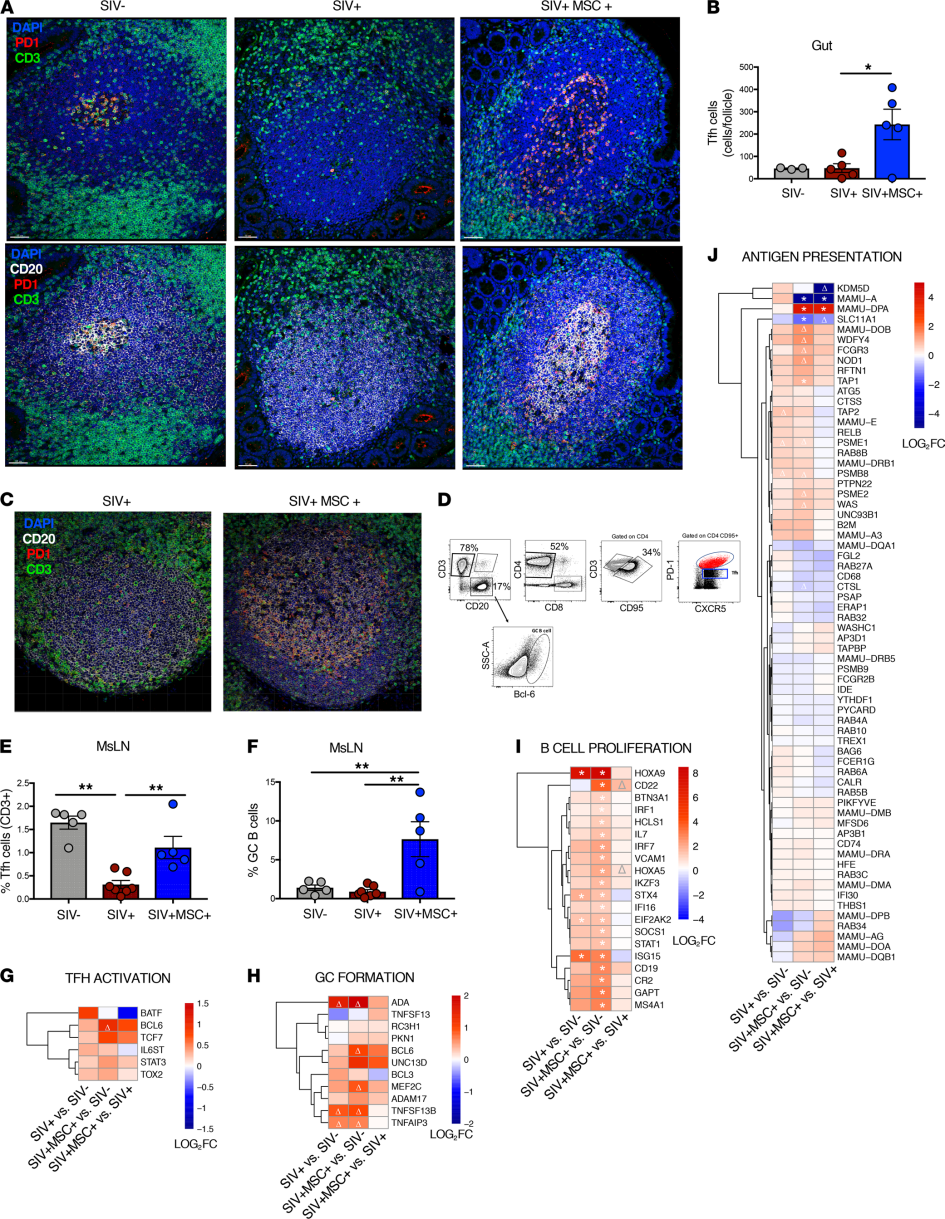
MSC administration leads to a robust germinal center and Tfh response in the gut-associated lymphoid tissue and periphery. (**A**) IHC analysis of cells expressing PD-1^+^, CD3^+^, and CD20^+^ within ileal lymphoid follicles from representative SIV^–^, SIV^+^, and SIV^+^MSC^+^ macaques. Gut sections show nuclei (blue), anti-CD20 (white), anti–PD-1 (red), and anti-CD3 (green) stains to highlight PD-1 expression on CD3-expressing cells in the lymphoid follicle. Magnification 20×. Scale bars: 50 μm. (**B**) Quantification of CD3^+^ PD-1^+^ cells per follicle. (**C**) Representative images of MsLN follicles of SIV^+^ and SIV^+^MSC^+^ animals. Magnification 20×. Scale bars: 50 μm. (**D**) Gating strategy for identification of Tfh cells and GC B cells. Cells were previously gated on lymphocytes, singlets, and live cells. (**E** and **F**) Percentage of Tfh (CD4^+^ CXCR5^+^) and GC B cells in MsLNs (SIV^–^ *n* = 5, SIV^+^ *n* = 7, and SIV^+^MSC^+^ *n* = 5). (**G–J**) Heatmaps display differential expression of genes associated with Tfh activation, GC formation, B cell proliferation, and antigen presentation. Significant (*FDR < 0.1) and trending (Δ *P* < 0.05) genes annotated. Data represent the mean (± SEM) for each time point. Significance was determined using 1-way ANOVA with Holm-Sidak post hoc testing for multiple comparisons (**C**, **D**, and **F**) or Spearman’s rank correlation with linear regression (**E**). *P < 0.05 and **P ≤ 0.01.

To determine the cellular mechanisms of SIV clearance from gut effector sites in MSC-treated animals, we investigated effects of MSC on the restoration of the gut lymphoid follicle structure and Tfh and B cells by IHC and immunophenotypic analyses. Interactions between Tfh cells and B cells within GCs are critical for promoting B cell maturation, antibody production, and long-lived plasma memory cell generation in lymphoid follicles and are associated with effective viral control during HIV and SIV infections (24). IHC analysis of intestinal tissues showed that MSC-treated animals had increased frequency of Tfh (CD3^+^ PD-1^+^) cells in the GCs of ileal lymphoid follicles (Figure 3, A and B, and Supplemental Figures 3A). Similarly, MsLNs had increased frequency of Tfh cells as detected by IHC and flow cytometric analysis (CD3^+^ CD4^+^ PD-1^int^ CXCR5^+^) (Figure 3, C–E). No significant difference was detected in the percentage of Tfh cells in the peripheral blood (Supplemental Figure 3B), suggesting that this effect was pronounced in the GALT. An increase in GC B cells (CD3^–^ CD20^+^ CXCR5^+^) was detected and corroborated with the transcriptional signature enriched for genes regulating Tfh activation, GC formation, B cell proliferation, and antigen presentation (Figure 3, F–J). Importantly, a marked increase in the gene expression of IL-7 was detected in SIV-infected MSC-treated animals compared with the SIV-negative controls but not in SIV-infected untreated animals. As an important activator of both T and B cell development and survival, IL-7 is essential for lymphocyte homeostasis as well as proliferation of naive CD4^+^ and CD8^+^ subsets (25, 26). A notable, nonsignificant (*P* = 0.03, FDR = 0.41) trend of increase in BCL6 expression was also observed in infected MSC-treated animals. BCL6 is a crucial transcription factor and master regulator of B cell maturation following cognate antigen stimulation as it activates the B cells to proliferate rapidly in response to T cell–dependent antigens and supports Ig class switch recombination, somatic hypermutation, and GC formation (27). BCL6 is likewise an essential transcription factor for the induction of Tfh cell differentiation and for supporting cellular homing to the GCs to facilitate B cell receptor affinity maturation (28). CD22 expression was also upregulated in MSC-treated animals and is known to regulate B cell survival and proliferation as well B cell homing to the GALT (29). Collectively, IHC, immunophenotypic, and transcriptomic analyses demonstrated that MSC treatment induced regeneration of the GC structure and supported the resident Tfh cells and B cells, contributing to effective antigen presentation and T and B cell interactions.

### GC restoration and B cell activation enhance anti-SIV antibody levels.

**Figure 4:**
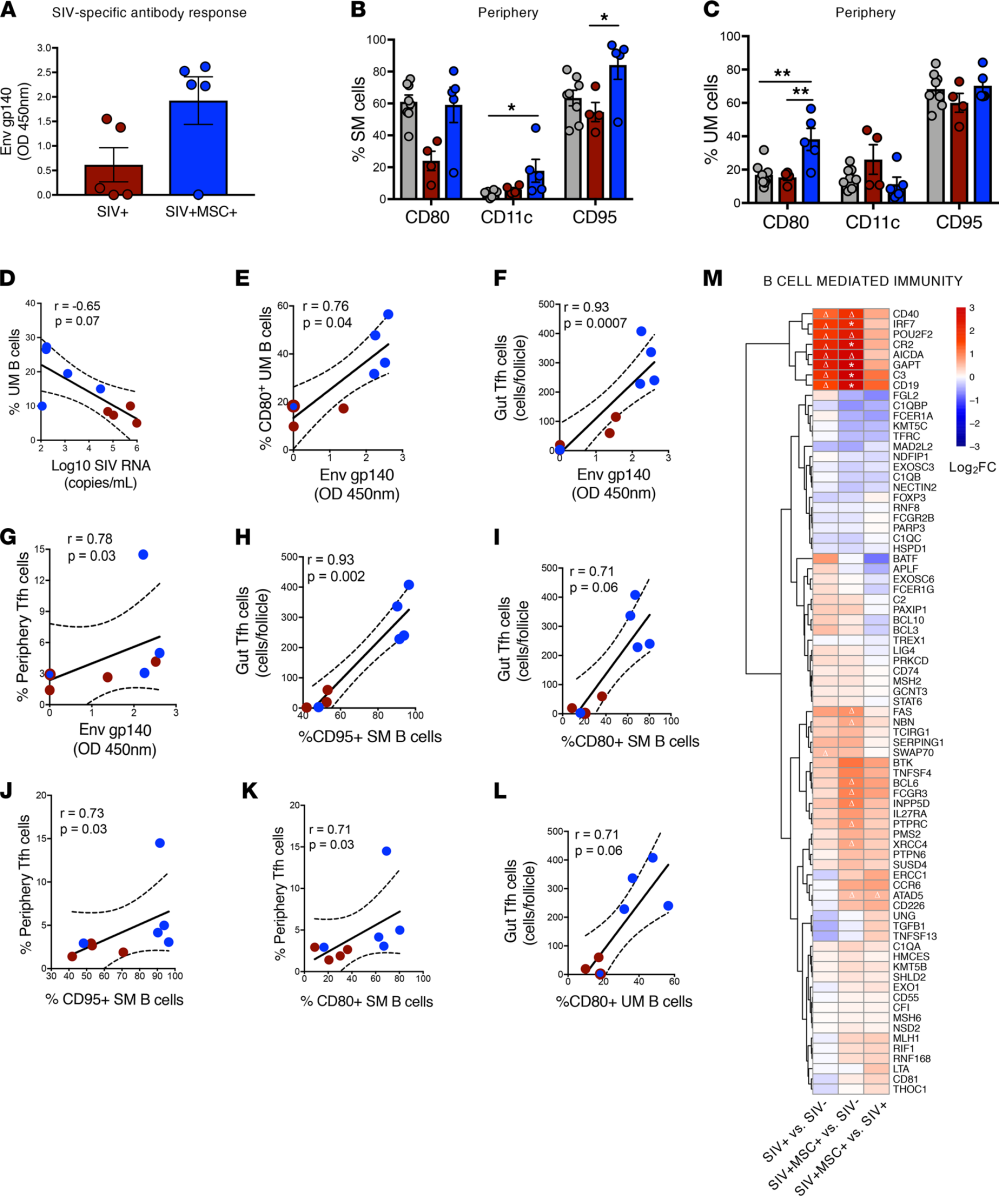
Elevated humoral and B cell response is associated with MSC-treated animals. (**A**) Measurement of IgG antibody using OD 450 nm measurement on gp140-coated ELISA plates (*P* = 0.06, NS), (SIV^+^ *n* = 5, SIV^+^MSC^+^ *n* = 5). (**B** and **C**) Activation status of the memory B cell subsets was measured by gating on CD80^+^, CD11c^+^, and CD95^+^ (SIV^–^ *n* = 8, SIV^+^ *n* = 4, SIV^+^MSC^+^ *n* = 5). (**D–L**) Spearman’s correlation was performed between activated B cell subsets, viral loads, and anti-gp140 antibody titers and the frequency of Tfh cells. Linear regression coefficients and *P* values are shown. Data represent 70 days after infection. (**M**) Heatmap displays differential expression of genes associated with B cell–mediated immunity. Significant (*FDR < 0.1) and trending (*P < 0.1; Δ < 0.05) genes annotated. Data represent the mean (± SEM) for each time point. Significance was determined using the Mann-Whitney *U* test (**A**), 1-way ANOVA with Holm-Sidak post hoc testing for multiple comparisons (**B** and **C**), or Spearman’s rank correlation with linear regression (**D–L**). *P < 0.05 and **P ≤ 0.01.

HIV and SIV infections cause follicular and GC damage in secondary lymphoid tissues and lead to impaired T and B cell interactions and suboptimal humoral responses (30, 31). Additionally, loss of activated memory B cells (CD21^–^ CD27^+^) is associated with disease progression (6, 32). We sought to investigate whether MSC-associated changes could enhance the antiviral humoral response. Measurement of the anti–SIV envelope gp140 antibody levels in peripheral blood samples of MSC-treated animals showed a trend of heightened SIV-specific antibody response (4/5 animals) compared with untreated SIV-infected animals (Figure 4A). We analyzed changes in the distribution of B cell subsets (Supplemental Figure 4A) and found no significant differences in the frequency of total (CD3^–^ CD20^+^), naive (CD3^–^ CD20^+^ CD27^–^ IgD^+^), and switched memory (CD3^–^ CD20^+^ CD27^+^ IgD^–^) B cells (Supplemental Figure 4, B and C) in MSC-treated animals compared with untreated controls. However, a significant increase in unswitched memory (CD3^–^ CD20^+^ CD27^+^ IgD^+^) B cells was detected in MSC-treated animals compared with SIV-infected animals and SIV-negative controls, suggesting an increased frequency of antigen-experienced B cells (Supplemental Figure 4C). As expected, our data showed that SIV infection resulted in the loss of activated switched memory B cells (expressing CD80, CD11c, and CD95). However, MSC treatment restored this activated B cell subset (Figure 4B) as well as CD80^+^ unswitched memory B cells (Figure 4C). The frequency of total unswitched memory B cells negatively correlated with viral loads (Figure 4D), whereas the frequency of CD80^+^ unswitched memory and Tfh cells (ileal and peripheral) positively correlated with the SIV-specific IgG response (Figure 4, E–G). In addition, ileal and peripheral Tfh cell levels positively correlated with CD95^+^ and CD80^+^ switched memory B cell percentages (Figure 4, H–L), suggesting that MSC-treated animals had effective T and B cell interactions in the GCs, supporting increased anti-SIV antibody production.

The mucosal transcriptomic analysis provided insights into the molecular mechanisms for enhanced anti-SIV antibody production in MSC-treated macaques. Significant expression of IRF7, GAPT, CR2, and CD19 genes regulating Ig class-switching, memory B cell development, and GC response were increased in MSC-treated animals compared with SIV-negative controls (Figure 4M). We also observed a trend of elevated CD40, AICDA, and ATAD5. Thus, administration of MSC to SIV-infected macaques restored the prevalence and function of Tfh cells and their interaction with B cells. This could promote the selection of SIV-specific B cells and enhance antibody response, leading to better viral clearance.

### Heightened SIV-specific CD8^+^ T cell response after MSC administration.

**Figure 5:**
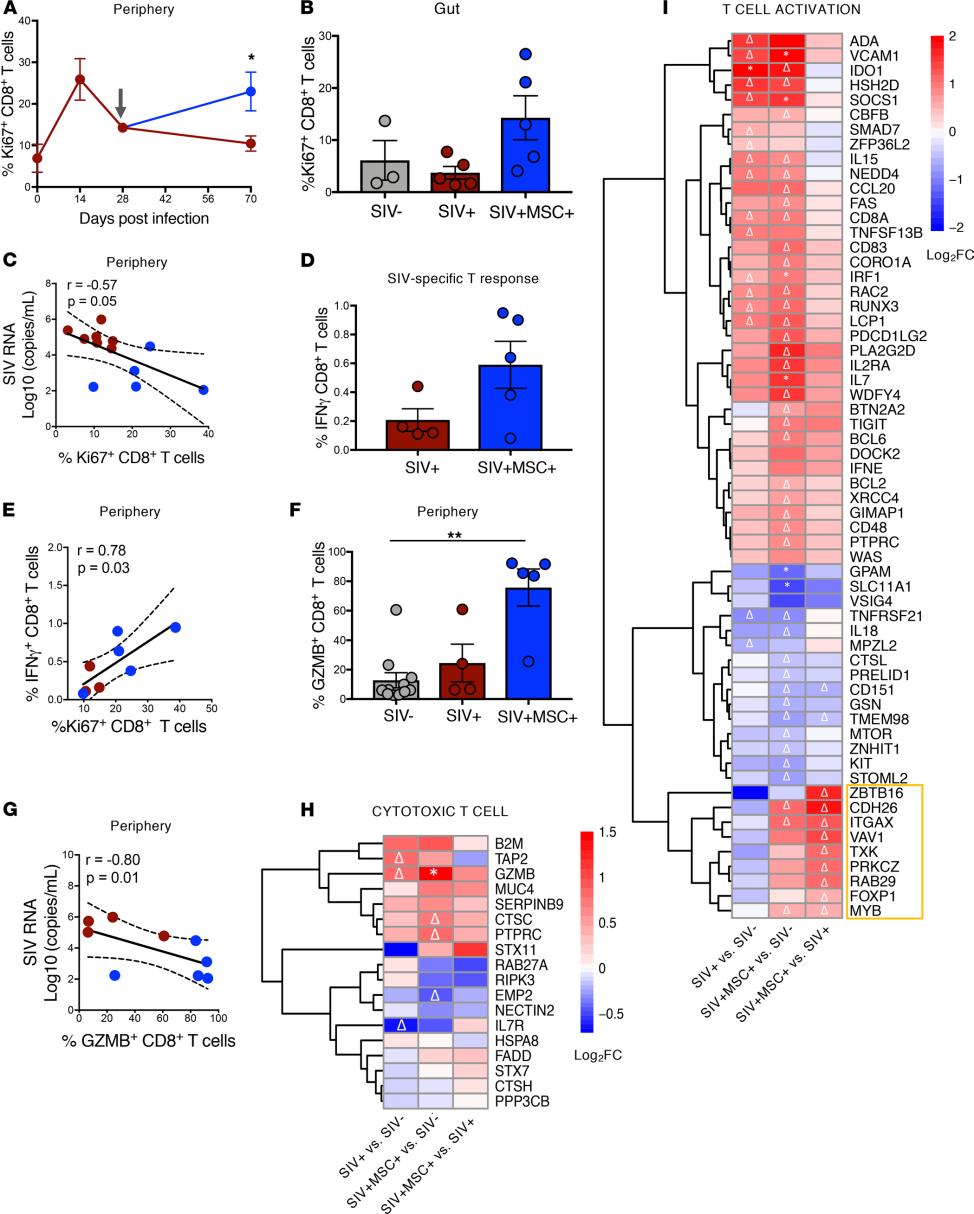
MSC promotes greater T cell proliferation and specific response to virus. (**A** and **B**) Percentages of proliferating Ki67^+^ CD8^+^ cells were determined by flow cytometry in the gut (SIV^–^ *n* = 3, SIV^+^ *n* = 5, and SIV^+^MSC^+^ *n* = 5) and periphery (SIV^+^ *n* = 7, SIV^+^MSC^+^ *n* = 5) at 70 days after infection and longitudinally, respectively. (**C**) Spearman’s correlation between proliferating CD8^+^ T cells and viral loads. Linear regression coefficients and *P* values are shown. (**D**) SIV-specific cellular response. Intracellular expression of IFN-γ in SIV-gag peptide–stimulated CD8^+^ (*P* = 0.29, NS) T cells (SIV^+^ *n* = 4, SIV^+^MSC^+^ *n* = 5). (**E**) Spearman’s correlation between SIV-specific IFN-γ CD8^+^ T cell response and frequency of proliferating CD8^+^ T cells (SIV^+^ *n* = 3 and SIV^+^MSC^+^ *n* = 5). (**F**) Frequency of granzyme B (GZMB) expression in CD8^+^ T cells (SIV^+^MSC^+^ vs. SIV^+^ is *P* = 0.06, NS) (SIV^–^ *n* = 11, SIV^+^ *n* = 4, and SIV^+^MSC^+^ *n* = 5). (**G**) Spearman’s correlation analysis relating the frequency of GZMB^+^ CD8^+^ T cells with plasma SIV viral loads (*P* = 0.08, NS) (SIV^+^ *n* = 4, SIV^+^MSC^+^ *n* = 5). (**H** and **I**) Heatmap displays differential expression of genes associated with T cell cytotoxicity and activation. Significant (*FDR < 0.1) and trending (*P < 0.1; Δ < 0.05) genes annotated. Data represent the mean (± SEM) for each time point. Significance was determined using the 1-way ANOVA with Holm-Sidak post hoc testing for multiple comparisons (**A**), the Mann- Whitney *U* test (**B** and **D**), Spearman’s rank correlation with linear regression (**C**, **E**, and **G**), or Kruskal-Wallis with Dunn’s post hoc testing (**F**). *P < 0.05 and **P ≤ 0.01. Gray arrow represents the first MSC administration.

We hypothesized that enhanced antiviral immunity during MSC treatment may include increased SIV-specific cytotoxic CD8^+^ T cell activity, contributing to viral reduction. Determination of the frequency of proliferating T cell subsets in PBMCs showed a significant increase in Ki67^+^ CD8^+^ T cells in MSC-treated animals (Figure 5A). A similar trend was observed in the gut (Figure 5B). However, no significant differences were observed in proliferating Ki67^+^ CD4^+^ T cells as well in the frequency of total CD4^+^ and CD8^+^ T cells (Supplemental Figure 5, A–D). The increased frequency of peripheral proliferating CD8^+^ T cells inversely correlated with plasma viral loads, suggesting an enhanced antiviral cellular response (Figure 5C). We determined SIV-specific CD8^+^ T cell responses in peripheral blood by measuring IFN-γ production after cell stimulation with SIV-gag peptides. A trend of increased percentages of SIV-gag–specific CD8^+^ T cell subsets was detected in the MSC-treated group (4/5 animals) compared with untreated controls (Figure 5D). The frequency of IFN-γ^+^ CD8^+^ T cells showed a significant positive correlation with proliferating CD8^+^ T cells in the periphery, suggesting that there was a clonal expansion of SIV-specific cytotoxic T cells (Figure 5E). Four out of the 5 MSC-treated animals also had a substantially higher frequency of CD8^+^ T cells expressing granzyme B (GZMB), a cytotoxic effector protease, and displayed a significant inverse correlation with plasma viral loads (Figure 5, F and G). This further supports an increased prevalence of SIV-specific CD8^+^ cytotoxic T cell activity in MSC-treated animals. The transcriptomic analysis identified molecular targets associated with the antiviral cellular response as well. Increased transcription of genes associated with T cell activation and cytotoxic response (including GZMB) was seen in MSC-treated animals compared with untreated animals and SIV-negative controls (Figure 5, H and I). Increased mucosal IL-7 and IL-7 receptor expression and activation of the IL-7 signaling pathway in MSC-treated animals can contribute to antiviral CD8^+^ T cell survival and expansion. Thus, MSC treatment resulted in an increase in the levels of virus-specific CD8^+^ T cells, leading to a heightened SIV-specific cellular response, which contributed to viral reduction.

### SIV-induced lipid metabolic dysregulation is reversed after MSC administration.

**Figure 6:**
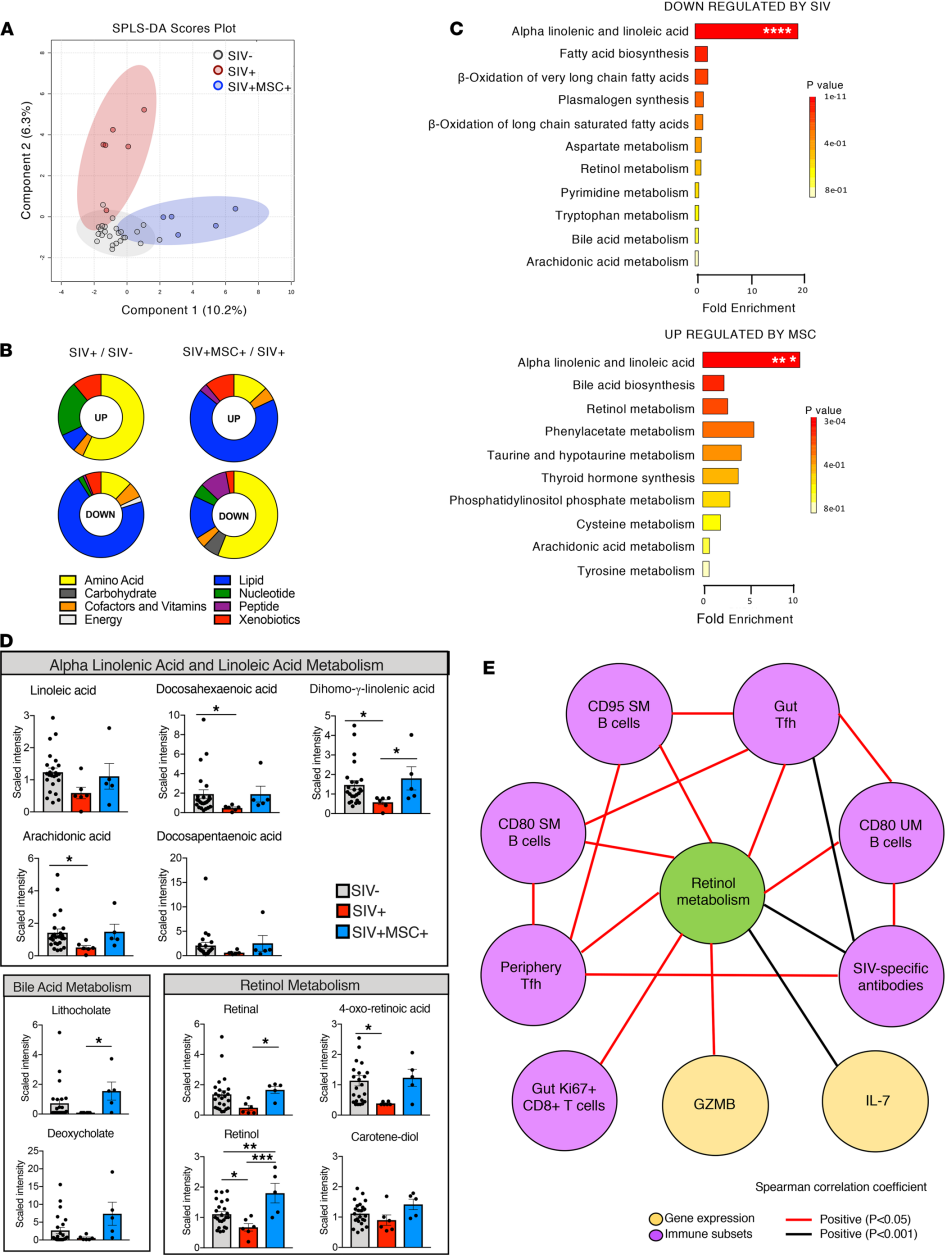
Immunometabolites associated with gut T cell trafficking, germinal center formation, B and T cell activation, and antiinflammatory response are elevated in MSC-treated macaques. (**A**) SPLS-DA plots showed a high degree of separation between groups (SIV^–^ *n* = 24, SIV^+^ *n* = 6, and SIV^+^MSC^+^ *n* = 5) at 70 days after infection. SIV^–^ group included preinfection longitudinal samples from SIV-infected animals in both SIV^+^ and SIV^+^MSC^+^ groups. (**B**) Pie charts showing the percentage of metabolites significantly upregulated and downregulated. (**C**) Altered metabolic pathways in SIV^+^ compared with SIV^–^ controls (*n* = 10) and SIV^+^MSC^+^ (*n* = 5) compared with SIV^+^ (*n* = 10) animals. The top 25 (FC ≥ ±1.5, *P* ≤ 0.05) plasma metabolites upregulated and downregulated were mapped to the appropriate KEGG identifiers and used as input for MSEA analysis using MetaboAnalyst software. Significance was tested using overrepresentation analysis with the hypergeometric test and reported *P* values shown after correction for multiple comparisons. The bar length shows fold enrichment. Pathways in blue highlight overlap between comparisons. (**D**) Metabolites belonging to alpha-linolenic and linoleic acid, bile acid, and retinol pathways (SIV^–^ *n* = 24, SIV^+^ *n* = 6, and SIV^+^MSC^+^ *n* = 5). (**E**) Significant correlations between retinol metabolism, Tfh, activated B cell subsets, SIV-specific response, proliferating gut CD8^+^ T cells, GZMB, and IL-7 plotted in network form. Significance was determined using Kruskal-Wallis with Dunn’s post hoc testing, 1-way ANOVA (linoleic acid, retinol, and carotene-diol), or Spearman’s rank correlation with linear regression (**E**). *P < 0.05, **P ≤ 0.01, ***P ≤ 0.001, and ****P < 0.0001.

Metabolic abnormalities are well-recognized in HIV-infected patients and are linked to inflammation, immune dysregulation, and microbial translocation (33). We hypothesized that MSC-induced enhancement of antiviral immunity and reversal of viral pathogenic effects would be reflected through changes in the host metabolomic profiles. Sparse partial least-squares discriminant analysis (SPLS-DA) of metabolic profiles revealed a distinct separation between MSC-treated and untreated control animals, indicating that MSC-associated changes were well-reflected in the cell metabolism and had a striking effect on the metabolic profiles (Figure 6A). A total of 127 metabolites were significantly altered (FC > 1.5; *P* < 0.05; *q* < 0.2) in untreated animals compared with SIV-negative controls, and 178 metabolites were altered in response to the MSC treatment compared with untreated controls (Supplemental Tables 1 and 2). HIV infection is also associated with biogenic amine perturbations (34) with elevated levels of amino acid–derived metabolites as well as decreased lipid levels that are not fully restored even after ART (33). The most pronounced metabolic changes in SIV infection included elevated levels of amino acid metabolism with a substantial decrease in lipid metabolism (Figure 6B). However, MSC treatment partially reversed this metabolic dysfunction by increasing lipid metabolite levels that were comparable to those in SIV-negative controls.

The most altered top 25 metabolites (upregulated and downregulated) were mapped to the appropriate Kyoto Encyclopedia of Genes and Genomes (KEGG) identifiers and used as input for metabolite set enrichment analysis (MSEA) using MetaboAnalyst software. The alpha-linolenic and linoleic acid pathway was significantly downregulated in SIV-infected animals compared with SIV-negative controls, but MSC treatment restored this critical antiinflammatory fatty acid pathway (Figure 6, C and D). This was evident through increased levels of linoleic acid, arachidonic acid, docosahexaenoic acid, docosapentaenoic acid, and dihomo-γ-linolenic acid (Figure 6D). Linoleic acid and alpha-linoleic acid belong to the omega-6 and omega-3 series of polyunsaturated fatty acids (PUFAs), respectively, and are known to induce systemic antiinflammatory pathways (35). Additionally, a notable enrichment of bile acid and retinol metabolites, including lithocholate, deoxycholate, retinal, retinol, 4-oxo-retinoic acid, and carotene-diol-2, were detected in MSC-treated animals compared with untreated SIV-infected controls (Figure 6D). While bile acid metabolism is essential for gut homeostasis, retinol metabolism plays an integral role in innate immunity and gut homing of activated B and T cells through augmented expression of α4β7 integrin and CCR9, respectively (36, 37). We used regression analysis to investigate a possible association of retinol metabolism and host immunity since these metabolites are also known to support GC generation and play a key role in B cell differentiation, class-switch recombination, and generation of antibody-secreting plasma cells (38). We found a network of significant correlations between retinoic acid (RA) and SIV-specific antibody levels, activated memory B cells, colon proliferating CD8^+^ T cells, and Tfh subsets from peripheral blood and tissue compartments as well IL-7 and GZMB expression (Figure 6E). Our findings suggest that elevated PUFA, RA, and bile acid metabolite levels in MSC-treated animals reflect a reversal of SIV-induced metabolic abnormalities and restoration of pathways associated with recruitment of activated lymphocytes for enhanced mucosal antiviral immunity and viral suppression.

## Discussion

In this study, we report for the first time, to our knowledge, the role of MSCs in modulating type I/II IFN signaling, leading to enhanced antigen presentation and the restoration of GCs as well as B and T cell interactions in the ART-naive, SIV-infected rhesus macaque model of AIDS. The rapid recovery of B cell follicle structure was associated with accumulation of Tfh cells, expression of IL-7, and virus clearance from effector sites. This provides a new opportunity and scientific rationale to leverage MSC treatment to enhance antiviral immunity, resolve virus-associated lymphoid follicle damage, and induce effective viral clearance in future multipronged HIV eradication strategies.

Our findings suggest that MSC treatment produces an immunophenotype resembling some of the characteristics of long-term HIV-infected non-progressors. HIV controllers have highly effective HIV-specific CD8^+^ and CD4^+^ T cell responses compared with HIV progressors and are reflective of GC integrity and function (9, 39, 40). Regulation of GC homeostasis and function is important for effective antiviral immunity and better clinical outcome in HIV infection. Our present data of viral reduction in peripheral blood and clearance of SIV RNA–positive cells from the extrafollicular zones in the GALT suggest that cytotoxic T cells have increased function for eradication of SIV-infected cells from MSC-treated macaques. We previously reported that cytolytic responses were predictive of mucosal immune recovery in SIV and HIV infections (41, 42). We found SIV RNA predominantly restricted to the GCs in lymphoid follicles of MSC-treated animals. The reticular viral staining pattern in gut lymphoid follicles suggested enhanced trapping of viral RNA on the follicular DC network, which would support better antigen presentation to B cells and stimulate effective Tfh interactions in the GC, leading to enhanced antibody production. The Tfh cells support B cell class switching, affinity maturation, and control GC development for the production of highly specific antibodies (43). Notably, we found an increased frequency of Tfh subsets in GALT and MsLNs that was associated with key regulators of Tfh differentiation in our gut transcriptomic dataset, including type I/II IFN signaling (44) and a trend of greater BCL-6 expression (28). The abundance of Tfh cells positively correlated with the SIV envelope–specific antibody response and activated memory B cell subset levels in MSC-treated animals, suggesting the reversal of pathogenic effects of SIV infection on Tfh cells and B cells (45). Importantly, recent studies showed that early Tfh recruitment and GC reactions were strongly associated with SIV viremia control in previously vaccinated rhesus macaques, suggesting that MSC administration may also be used in vaccine approaches to stimulate protective responses (46). Collectively, our data suggest that enhanced antigen presentation in the GALT of MSC-treated animals led to increased levels of anti-SIV antibodies and SIV-specific CD8^+^ T cells that resulted in viral reduction.

In our study, MSC treatment of SIV-infected macaques resulted in the induction of activated memory B cells, elevated SIV envelope–specific antibodies, and increased cytotoxic CD8^+^ T cell functions with related gene signatures. This led to decreased plasma viral loads and substantial clearance of SIV RNA–positive cells from gut lamina propria. The presence of virus-specific neutralizing antibodies, cytotoxic CD8^+^ T cells, and preservation of CD4^+^ T cells in long-term HIV-infected non-progressors highlight the mechanisms of viral control and are reflective of effective antigen presentation in the well-maintained lymphoid follicles (47, 48). Transcriptomic analyses in previous studies have identified gene expression and networks related to B cell and CD8^+^ T cell signatures correlating to protection or suppression of HIV in long-term HIV-infected non-progressors and in vaccine studies (9, 11). Our study was of short-term duration and involved MSC administration over 6 weeks because we sought to identify the early and immediate effects of MSCs in chronic SIV infection independent of ART. Together, our findings identified the ability of MSC to induce regeneration of lymphoid follicles, activate humoral and cellular immunity and related mucosal gene signatures, and cause viral reduction.

Transcriptomic analysis of MSC treatment–associated gene expression changes revealed the dominant impact of genes in host defense response to the virus and activation of type I/II IFN signaling. The MSC-associated gut mucosal transcriptomic signature included immune mediators, such as GZMB, TRIM5-α, IRF1, IRF7, and PML. Notably, GZMB secreted by NK and CD8^+^ T cells induces apoptosis of virally infected targets. TRIM5α, a known HIV host-restriction factor, blocks HIV reverse transcription. Increased expression of TRIM5α has been reported in HIV elite controllers compared with typical progressors (49). In the MSC-induced mucosal gene expression signature, IL-7 transcript levels were significantly upregulated, indicating the contribution of IL-7 to the restoration of mucosal CD4^+^ T cells and increase frequencies of GZMB-positive proliferating CD8^+^ T cells. Increased IL-7 receptor expression in these samples further supports the major role of the IL-7/IL-7R signaling pathway in T cell functionality. Previous studies have demonstrated that IL-7 therapy revives HIV-specific cytotoxic T cell activity, promotes Tfh and B cell interactions, and contributes to viral clearance (50, 51). Our prior findings also suggest that a threshold of more than 50% CD4^+^ T cell restoration was sufficient for generating polyfunctional HIV-specific T cells (42). Our current findings demonstrated that early effects of MSC treatment on mucosal lymphoid follicle structure and function were at the threshold of positively affecting antiviral immunity, viral reduction, and CD4^+^ T cell recovery. Thus, MSCs may initiate a trajectory of antiviral immunity and gut mucosal restoration, leading to long-term viral control.

Dysregulation of lipid metabolism has been well documented in HIV and SIV infections (18, 33). Transcriptomic changes in viral infections and diseases are reflected in altered metabolomic profiles. We found that MSC-treated animals had increased levels of PUFA metabolites, including linoleic acid, dihomo-γ-linolenic acid, docosahexaenoic acid, and arachidonic acid, which play a role in immunomodulation. These metabolites display antibacterial, antifungal, and antiviral activity in the host as well as stimulation of the production of resolvins, lipoxins, protectins, and maresins that greatly reduce inflammation and subsequent tissue damage (52). Dihomo-γ-linolenic acid and arachidonic acid, in particular, are precursors for the synthesis of prostaglandins and eicosanoids, which mediate the effects of type I IFNs during antiviral immune response (52). It has been previously reported that linoleic acid metabolism positively correlated with CD4^+^ T cell frequency in HIV-infected patients, whereas its metabolic dysregulation was strongly associated with disease progression and mortality (53). Finally, RA and bile acid metabolism, known to regulate gut lymphocyte homing and mucosal homeostasis, were significantly boosted in MSC-treated animals. RA enhances the expression of α4β7 integrin and CCR-9 on activated B and T cells to facilitate migration from the peripheral blood to GALT (37); bile acid metabolites have been reported to reduce inflammation through modulation of T cell function and the Th-17/Treg balance in the gut (54). RA is also involved in T and B cell proliferation, class switch recombination, and GC reaction. RA increases CD40 expression on DCs, promotes B cell migration to GCs, and affects follicular DC network function to increase antigen presentation and antibody production (38). Interestingly, we identified increased expression of CD22 in the B cell proliferation pathway of SIV-infected MSC-treated but not untreated animals. CD22 has been shown to regulate gut-specific homing as CD22-ligand is exclusively expressed in the high endothelial venules of gut Peyer’s patches (55). Thus, an increase in immunomodulatory metabolites observed in peripheral blood reflects a heightened immune response as well as restoration of lymphoid tissue function in MSC-treated animals.

One of the limitations of our study is the relatively small number of nonhuman primates per each experimental group. Nonhuman primate studies are extensive in their scope and tend to have smaller numbers of animals per group compared with mouse studies. However, the strengths and benefits of this model far outweigh the limitation in animal numbers. The structure, function, and cell composition of the nonhuman primate gut and lymphoid tissues are strikingly similar to those in humans. Additionally, SIV infection recapitulates HIV pathogenic effects. The viral infection kinetics, mucosal damage, immune dysfunction, and impaired molecular networks are well characterized and highly suitable to investigate MSC biology and functions in the repair of the virally inflamed tissue microenvironment. Another limitation was the short duration and lack of long-term follow-up on the outcomes. Our study was designed to capture the early effects of MSC therapy in the lymphoid compartments of SIV-infected animals and to collect gut mucosal lymphoid tissue for extensive and integrated analyses at 6 weeks after the start of MSC infusion. This enabled us to identify early changes in the peripheral and GALT compartments and fill the knowledge gap in this field. Finally, many of our conclusions regarding MSC effects derive from the experimental endpoint because longitudinal samples of gut tissue are largely inaccessible while the study is in progress. However, our integrated analysis approach for flow cytometric, IHC, transcriptomic, and metabolomic data has highlighted beneficial effects of MSC in chronic SIV infection and supports the need for a long-term follow-up investigation.

In summary, our data showed that systemic MSC infusion induced significant virus reduction as detected by decreased plasma viral loads and clearance of virus-positive cells from gut effector sites. The enhanced antiviral response resulting from the regeneration of gut GCs of the lymphoid follicles and restoration of Tfh and B cell compartments allowed an effective antigen presentation through corralling of the virus into the lymphoid follicles and T and B cell interactions. Transcriptomic analysis identified molecular pathways and gene signatures supporting immune-mediated viral reduction and mucosal recovery in MSC-treated animals that included type I/II IFN signaling. Enhanced expression of genes regulating GC formation, T and B cell activation, and host innate antiviral defenses strongly suggests that MSCs may directly or indirectly modulate these pathways during SIV infection. Metabolomic analysis further demonstrated that MSC administration reversed SIV-induced dysregulation of lipid metabolism and restored the retinol pathway to affect host immunity. Our findings provide the rationale for investigating the role of MSC treatment in HIV infection to boost host antiviral immunity and offer an opportunity to complement current therapeutic approaches for an innovative, multipronged HIV cure strategy.

## Methods

### Study design and sample collection.

Thirty-five rhesus macaques (*Macaca mulatta*) ages 4 to 13 years were obtained from the California National Primate Research Center (CNPRC; Supplemental Table 3). Seventeen animals were i.v. infected with 1000 TCID50 of SIVmac251 on day 0. Five animals were treated with allogeneic MSCs from a young rhesus donor. A dosage of 5 × 10^6^ cells/kg was administered on days 28, 42, and 56 after SIV infection. All SIV-infected animals were euthanized and necropsied at day 70 after infection. Peripheral blood samples, colonic biopsies, and rectal swabs were collected before and throughout SIV infection to provide longitudinal data. In addition, samples from 18 SIV-negative animals were included for analyses in the present study to serve as controls. A subset of control animal samples was selected for analyses based on quality and availability for each analysis. The present study was performed in accordance with the recommendations of the Public Health Services Policy on Humane Care and Use of Laboratory Animals. All animal procedures were performed according to a protocol approved by the Institutional Animal Care and Use Committee (IACUC) of the University of California, Davis. Approved sedatives, anesthetics, and analgesics were used during handling and surgical procedures to minimize pain or discomfort to animals. Animals were euthanized in accordance with the American Veterinary Medical Association Guidelines for the Euthanasia of Animals.

### MSC culture and infusions.

MSCs were obtained from the bone marrow from an infant rhesus macaque at the CNPRC, University of California, Davis, as previously reported (56). Cells were expanded for phenotypical and functional characterization by flow cytometry and differentiation. Cells (1 × 10^6^) were cultured to the third passage, stained, and selected for all positive (CD1a, CD14, CD29, CD34, CD58, CD71, CD90, CD166) MSC markers. Cell differentiation potential was tested using osteogenic, adipogenic, and chondrogenic differentiation medium (Lonza) following the manufacturer’s recommendations and established procedures (56, 57). Cells were washed and tested for viability, sterility, mycoplasma, endotoxin, and potency and cryopreserved using a controlled-rate cryopreservation protocol that provides 90% or greater viability when cells are thawed. Prior to administration, preselected and characterized MSCs were thawed and plated in T175 flasks and cultured in complete media (DMEM, Thermo Fisher Scientific) supplemented with 20% heat-inactivated FBS (Thermo Fisher Scientific), 1% GlutaMAX (Thermo Fisher Scientific), and 1% penicillin-streptomycin (Thermo Fisher Scientific) until they reached approximately 80% confluency. Cells were harvested (0.05% trypsin, Thermo Fisher Scientific), washed in PBS, and resuspended in saline at a final concentration of 10 × 10^6^ cells/mL. Cell preparations had more than 85% cell viability and were tested for *mycoplasma* sp. (Gelantis) and LPS (Thermo Fisher Scientific) contamination.

### Viral load measurements.

Real-time reverse-transcription PCR (real-time RT-PCR) was performed to determine the levels of SIV-RNA loads in plasma and gut tissue samples as previously described (41).

### Flow cytometry analysis.

Flow cytometric analysis was performed to determine the distribution of T cell subsets and their activation status in the peripheral blood and gut. Cells were immunostained with the following antibodies: anti–CD45-Alexa Fluor700 (BD Biosciences, D058-1283), anti–CD3-Pacific Blue (BD Biosciences, SP34-2), anti–CD4-PerPCy5.5 (Biolegends, OKT4), and anti–CD8-Ped594 (Biolegends, RPA-T8). Cells were permeabilized with Cytofix/Cytoperm kit (BD Biosciences) and stained with anti–Ki67-Alexa Fluor 488 (Fisher Scientific, B56). For characterization of Tfh and cytotoxic T cells in peripheral blood, cells were immunostained for cell surface markers using anti–CD3 Alexa Fluor 700 (BD Biosciences, SP-234), anti–CD4 BV650 (BD Biosciences, L200), anti–CD8 PerCPCy5.5 (BD Biosciences, SK1), anti–CD95 APC (BD Biosciences, DX2), anti–CXCR5 BV421 (Invitrogen, MU5BEE), and anti–PD-1 PECy7 (Biolegend, EH12.2H8); and for intracellular markers using anti–GZMB PECF594 (BD Biosciences, GB11).

For immunophenotyping of Tfh in MsLN, cells were immunostained with anti–CD3 Alexa Fluor 700 (BD Biosciences, SP-234), anti–CD4 BV650 (BD Biosciences, L200), anti–CD8 BV510 (BD Biosciences, SK1), anti–CD20 BV421 (Biolegends, 2H7), anti–CD95 BUV737 (BD Biosciences, DX2), anti–CXCR5 PE (Invitrogen, MU5UBEE), and anti–PD-1 PECy7 (Biolegend, EH12.2H8).

For B cell immunophenotyping, PBMCs were immunostained with anti–CD11c BV650 (BD Biosciences, S-HCL-3), anti–CD20 APC-H7 (BD Biosciences, L27), anti–CD21-PECy7 (BD Biosciences, B-ly4), anti–CD27-PacBlue (Biolegend, M-T7271), anti–CD80 PE (BD Biosciences, L307-4), anti–IgD-FITC (Southern Biotech, 2030-02), and anti–CD95-APC (BD Biosciences, DX2).

Dead cells were excluded from each analysis using the LIVE cell stain kit (Invitrogen). Data were analyzed using FlowJo (v10.4.1) and all reagents are listed in Supplemental Table 4.

### SIV-specific T cell response.

PBMCs were suspended in RPMI media (Invitrogen) and rested for 2 hours. One million cells were stimulated in vitro with phorbol 12-myristate 13-acetate and ionomycin or with overlapping peptides from SIV p27 protein for 9 hours at 37°C as previously described (58). After incubation, cells were washed and immunostained with Alexa Fluor 700–conjugated anti-CD3 (BD Biosciences, SP-234), BV650-conjugated anti-CD4 (BD Biosciences, L200), and PerCyP-Cy5.5–conjugated anti-CD8 (Biolegend, OKT4). Cells were washed, permeabilized using a Cytofix/Cytoperm Kit (BD Biosciences), and intracellularly immunostained with PE-Cy7–conjugated anti–IFN-γ (BD Biosciences, B27) and Alexa Fluor 488–conjugated anti–TNF-α (Invitrogen, Mab11).

### IHC.

Gut tissue samples were fixed in 4% paraformaldehyde and paraffin-embedded. Tissue sections (5 μm) were treated with antigen retrieval buffer at 100°C (DAKO Target Antigen Retrieval Solution pH 6.1, S2375), blocked for 1 hour at room temperature, and incubated with the following primary antibodies: anti-CD20 (monoclonal mouse anti-human; 1:100; DAKOCytomation, M0755), anti-CD3 (polyclonal rabbit anti-human; 1:100; DAKOCytomation, A0452), and anti–PD-1 (goat anti-human; 1:50; R&D Systems, Alexa Fluor 1086). After overnight incubation at 4°C, slides were washed and stained with secondary antibodies: Alexa Fluor 488 donkey anti-rabbit (Invitrogen, A21206), Alexa Fluor 555 donkey anti-goat (Invitrogen, A21432), and Alexa Fluor 647 donkey anti-mouse (Invitrogen, A31571) for 1 hour at room temperature. Cell nuclei were visualized using DAPI nuclear counterstain (Invitrogen, D1306) and by mounting slides using ProLong Gold Antifade Mountant (Thermo Fisher Scientific).

### Confocal microscopy and image analysis.

Cells expressing CD3 and PD-1 were detected and quantitated. The *Z*-stacked, 3D images of ileal lymphoid follicles were captured using a 20×/0.75 oil objective (HC PL APO CS2) on a confocal microscope (Leica TCS SP8 STED 3X) with 0.2 μm optical slices and 2048 × 2048 pixel resolution. The raw.lif files were then deconvoluted using the CMLE algorithm with Huygens Professional software (Scientific Volume Imaging, http://svi.nl) and converted to.ims files for compatibility with Bitplane Imaris 8.2.1 software. We delineated each region of interest (average of 3 B cell follicles based on CD20 expression) and quantified total CD3^+^ PD-1^+^ cell counts using Imaris software and MATLAB commands.

### ELISA.

Plasma samples from SIV-infected animals at day 70 after infection were assayed for the presence of anti-SIVgp140 antibodies. ELISA plates were coated with recombinant SIVgp140 of SIVmac239 (NIH AIDS reagent program) for 1 hour at 37°C, washed with PBS Tween 20, and blocked. Plasma samples were serially diluted with a blocking buffer and applied to the ELISA plate, incubated for 2 hours at 37°C, and then plates were washed. HRP-conjugated anti-rhesus IgG antibody (Nordic MUbio, GAMon/IgG(Fc)/PO) was added to the ELISA plates and incubated for 1 hour at 37°C. Sequentially, TMB substrate (Sigma-Aldrich) and stop solution were added and the absorbance at 450 nm per well was read in a microplate reader.

### In situ RNA hybridization analysis.

SIV RNA–positive cells in gut and lymph node tissues were detected and localized by using an RNAscope assay (Advanced Cell Diagnostics, ACD) as previously reported (20). Briefly, FFPE tissue sections were deparaffinized and processed according to the RNAscope 2.5 HD Detection Reagent-RED user manual. Tissue sections were reacted with RNAscope probes (ACD) that included control probes RNAscope 2.5 Duplex Positive Control Probe (Hs) PPIB-C1/POLR2A-C2 (ACD, 321641) and RNAscope Negative Control Probe DapB (ACD, 310043) and the V-SIVmac251gag probe (ACD, 488091). Slides were imaged using an Olympus BH-2 bright-field microscope at 10× and 20× magnification. Vignette artifacts were removed from all images with the Photoshop lens correction feature.

### Combined in situ RNA hybridization and IHC.

To detect SIV RNA in PD-1–expressing T cells, a combination of RNAscope assay (SIV RNA detection) and IHC assay (PD-1 and CD3 detection) was used that detect both the SIV-RNA hybridized probe and fluorophore-labeled protein targets. Tissue sections (5 μm) were deparaffinized and hybridized with the V-SIVmac251gag probe (ACD, 488091). Control probes RNAscope 2.5 Duplex Positive Control Probe (Hs) PPIB-C1/POLR2A-C2 (ACD, 321641) and RNAscope Negative Control Probe DapB (ACD, 310043) were included, and the assay was performed according to the manufacturer’s instructions. After the final chromogenic stain with RNAscope 2.5 RED reagent, tissue sections were rinsed and placed in a blocking solution containing 15% donkey serum and 1% FcR blocking antibody for 1 hour. Primary antibodies CD3 (1:200) (DAKO, A0452) and PD-1 (1:50) (R&D Systems, AF1086) were applied overnight at 4°C. Secondary antibodies PD-1, donkey, anti-goat, Alexa Fluor 647 (Invitrogen, A31571) and CD3, goat, anti-rabbit, Alexa Fluor 488 (Invitrogen A21206) were applied (1:400) for 1.5 hours followed by DAPI staining to visualize cell nuclei. Tissue sections were imaged using 20× and 100× oil objectives on a Leica TCS SP8 STED 3× confocal microscope with 2048 × 2048 pixel resolution. Huygens Professional software performed image deconvolution prior to final processing and image capture with Imaris v8.2.1.

### RNA extraction and sequencing.

Total RNA was isolated from ileum using the QIAGEN RNeasy Mini Kit (74106) and treated with DNase (QIAGEN RNase-Free DNase Kit, 79254). Samples possessed integrity scores above the 6.0 requirement with an average RNA integrity of 7.5. Barcoded sequencing libraries were prepared using the QuantSeq FWD kit (Lexogen) for multiplexed sequencing according to the manufacturer’s recommendations using the unique device identifier adapter and unique molecular identifier Second-Strand Synthesis modules (Lexogen). Fragment size distributions were verified with microcapillary gel electrophoresis on a LabChip GX system (PerkinElmer). Libraries were quantified on a Qubit fluorometer (Invitrogen), pooled in equimolar ratios, and sequenced. Finally, an Illumina HiSeq 4000 system generated approximately 30 million single-end reads with 100 bp lengths.

### Transcriptome alignment and differential gene expression analysis.

Briefly, read preprocessing was performed using Trimmomatic (0.36) to remove low-quality bases (Q20 cutoff), fragments with suboptimal length (bp < 50), and adapter sequences (Illumina-TruSeq2). Reads were aligned to the *Macaca* *mulatta* genome (Mmul_10) and quantified using STAR (v2.7.3a) (59). EdgeR (v3.28.1) software identified significant (FC > ±1.5, FDR < ±0.1) DEGs using R (v 3.6.1) and RStudio (60). Expression analysis was visualized with volcano plots and heatmaps generated by R in RStudio. The online platform Metascape (https://metascape.org) performed pathway and protein-protein interaction enrichment analysis using DEG input.

### Global metabolic profiling.

Metabolomic profiling in plasma samples was performed (Metabolon Inc.). Samples were processed using the automated MicroLab STAR system (Hamilton Company) as previously described (18). Differences in metabolite levels between groups were analyzed using a 2-tailed Mann-Whitney *U* test with FDR correction and considered significant when the *P* value was 0.05 or less and the FDR-corrected *P* value (*q* value) was 0.2 or less. MSEA was performed using MetaboAnalyst software (61).

### Statistics.

Statistical analyses were performed with GraphPad Prism software (v.7) for all immune analyses. Data normally distributed (Gaussian distribution) according to a Shapiro-Wilk test were analyzed using ANOVA; data non-normally distributed (non-Gaussian distribution) were analyzed using a Kruskal-Wallis test. Experimental groups were compared using the Mann-Whitney *U* test. All virological, cellular, and tissue analyses utilized a 2-sided test and were considered statistically significant for a *P* value of 0.05 or less. Paired correlations using immune data were assessed with a Spearman’s test using a 2-sided analysis. RNA-Seq data were analyzed for differential gene expression using the EdgeR (v1.22.2) package with a negative binomial distribution model of gene counts. The Benjamini-Hochberg (BH) method was used to control for the FDR of differential expression and Spearman’s correlation analyses were used when multiple comparisons were calculated in R. Metascape biological pathway enrichment calculated *P* values based on the accumulative hypergeometric distribution and corrected for multiple testing using the BH method. Terms with a *P* value less than 0.01, a minimum count of 3, and an enrichment factor greater than 1.5 (the enrichment factor is the ratio between the observed counts and the counts expected by chance) were clustered based on membership similarities. Kappa scores were used as the similarity metric when performing hierarchical clustering on enriched terms, and sub-trees with a similarity of *t* greater than 0.3 were considered a cluster. The most statistically significant pathway term within each group was selected to represent the whole cluster.

### Data and material availability.

The raw and processed next-generation sequencing data sets have been uploaded to NCBI’s Gene Expression Omnibus (GSE171635).

### Study approval.

All nonhuman primates in this study were maintained according to the guidelines of the IACUC of University of California, Davis, based on an approved protocol (#20136).

## Author contributions

SD, LAH, and AK designed the study and experimental plan; AFT and AK provided MSCs and their characterization. LAH, MGW, AK, SSI, SRE, CJWL, AM, and JA provided primary data. BB, CJWL, CSR, and MGW performed gene expression and metabolomic analyses. SD, MGW, and CJWL wrote the manuscript with input from CSR, AK, SSI, and AFT.

## Supplementary Material

Supplemental data

## Version 1 05/20/2021

In-Press Preview

## Version 2 06/22/2021

Electronic publication
