# Supplementary material for: Gut germinal center regeneration and enhanced antiviral immunity by mesenchymal stem/stromal cells in SIV infection
Source: JCI Insight. 2021 Jun 22;6(12):e149033. doi: 10.1172/jci.insight.149033 (PMC8262475; doi:10.1172/jci.insight.149033)
Supplement: Supplemental data [file jciinsight-6-149033-s047.pdf]

SUPPLEMENTARY MATERIALS

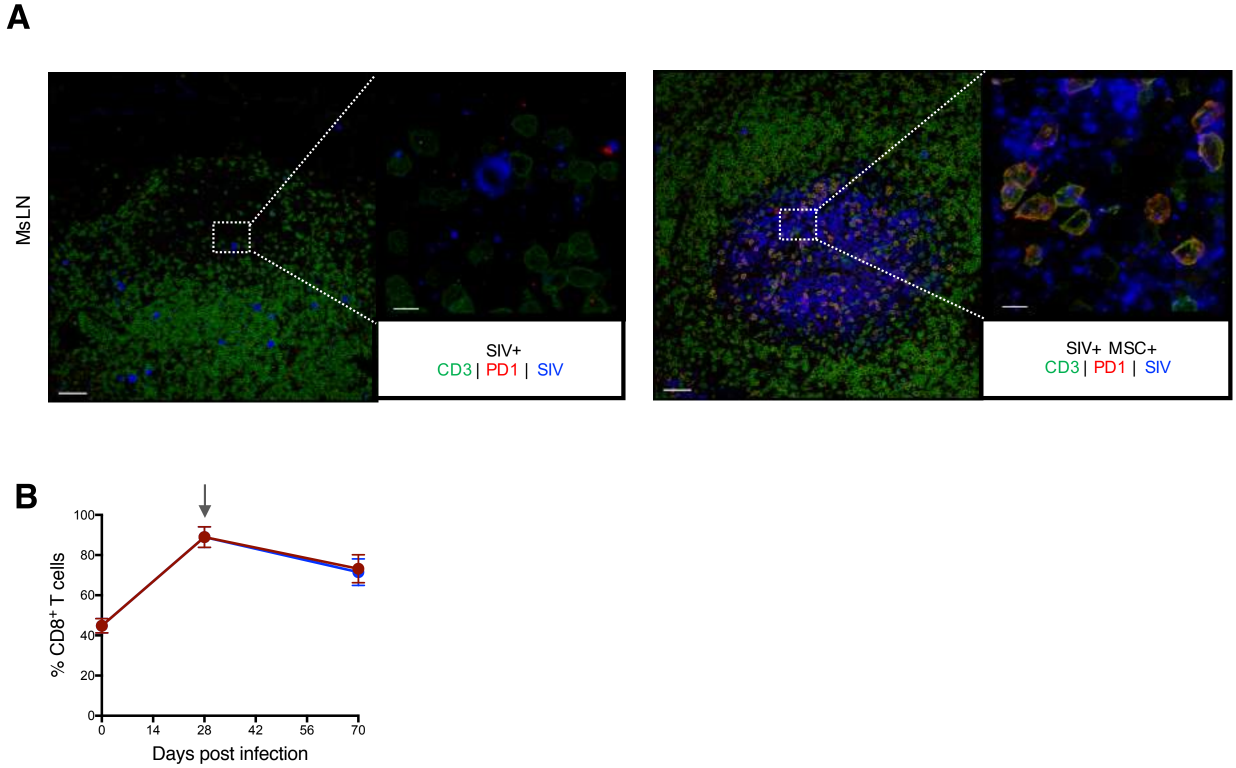

Supplementary Figure 1. Detection of viral loads in the periphery and localization in the tissue. (A) Dual in situ RNA hybridization and immunohistochemistry analysis of GC of the MsLN of SIV+MSC+ and SIV+, respectively, showing viral RNA (blue), T cells (green) and PD-1 (red). Magnification 20x and 100x. Scale bars 20µm and 50µm. **(B)** Longitudinal percentage of CD4+ T cells in the gut (SIV+ n = 7 and SIV+MSC+ n = 5). Data represent the mean (± SEM) for each time point. Grey arrow represents the first MSC administration.

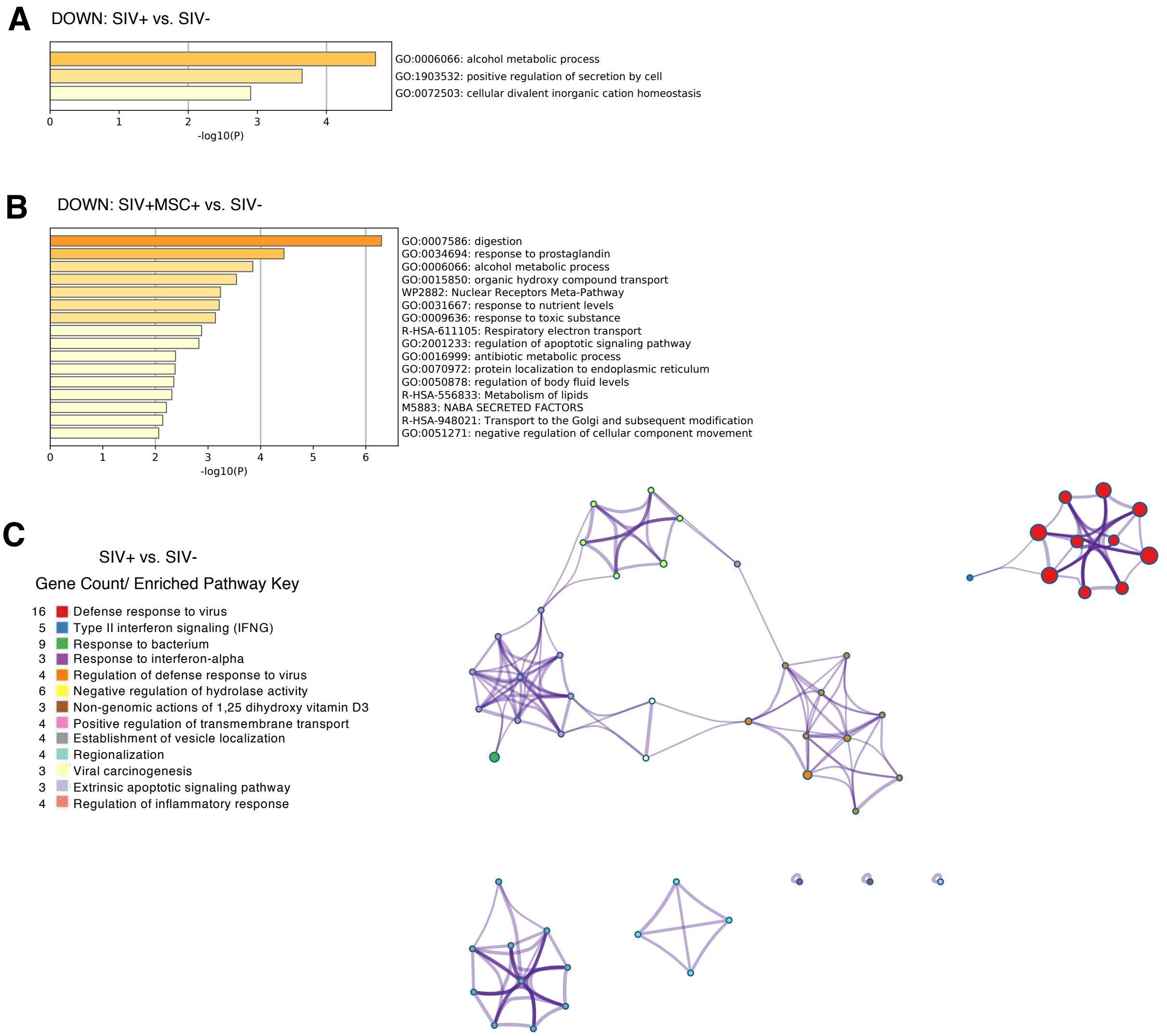

D

SIV+ vs. SIV-

MCODE	Pathway/Description	Log10(P)
1	IFN-α/β signaling	-26.3
	Type-1 IFN signaling	-24.9
	Cellular response to type-1 IFN	-24.9
2	ISG15 antiviral mechanism	-10.4
	Antiviral mechanism by IFN-stimulated genes	-10.2
	IFN signaling	-8.6
3	Allograft rejection	-7.5
	Peptide-ligand binding receptors	-6.4
	Positive regulation of transmembrane transport	-6.4

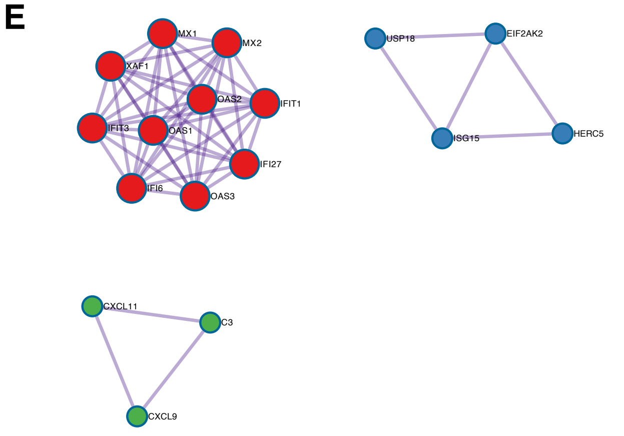

Supplementary Figure 2. Gut mucosal gene expression signature associated with MSC-treated SIV infected macaques. (A and B) Bar graphs show enriched down-regulated biological

pathways detected where P represents the Benjamini-Hochberg FDR. **(C)** Network analysis showing term enrichment (node size based on gene count) and interactions (edges) between the top 20 detected biological pathways (color). **(D)** Table displays enriched MCODE modules as determined by a protein-protein interaction analysis. **(E)** Expressed proteins with known physical interactions, associations and biological regulation from enriched modules. All analyses and figures were generated through Metascape.org. Significant down-regulated DEGs from SIV+MSC+ vs. SIV- and SIV+ vs. SIV- animals were used as input to evaluate the magnitude of mucosal antiviral immunity following MSC administration.

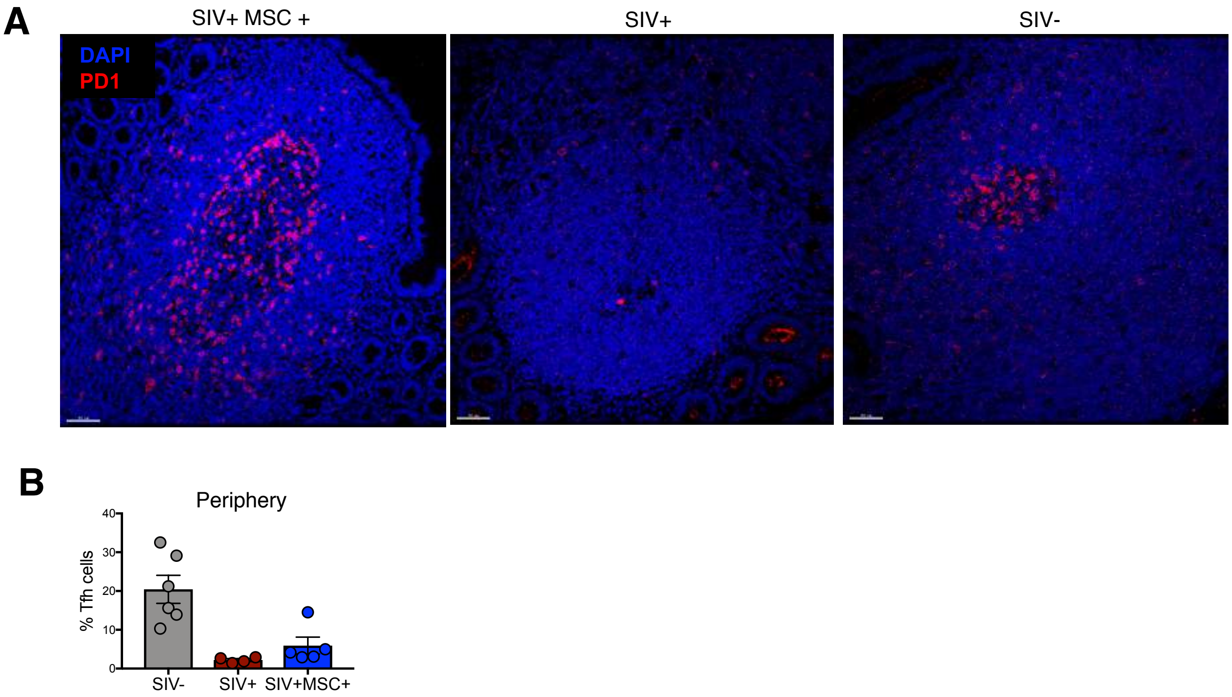

Supplementary Figure 3. Detection of PD-1+ T expression in gut lymphoid follicles (A) Immunohistochemical analysis of PD-1+ expressing cells within ileal lymphoid follicles from representative SIV-, SIV+ and SIV+MSC+ macaques at 70 dpi. Gut sections show nuclei (blue), and anti-PD-1 (red) in the lymphoid follicle. Magnification 20x. Scale bars 50µm. **(B)** Percentage of Tfh (CD4+ CXCR5+) determined by flow cytometry in the peripheral blood (SIV-=6, SIV+n=4, SIV+MSC+n=5).

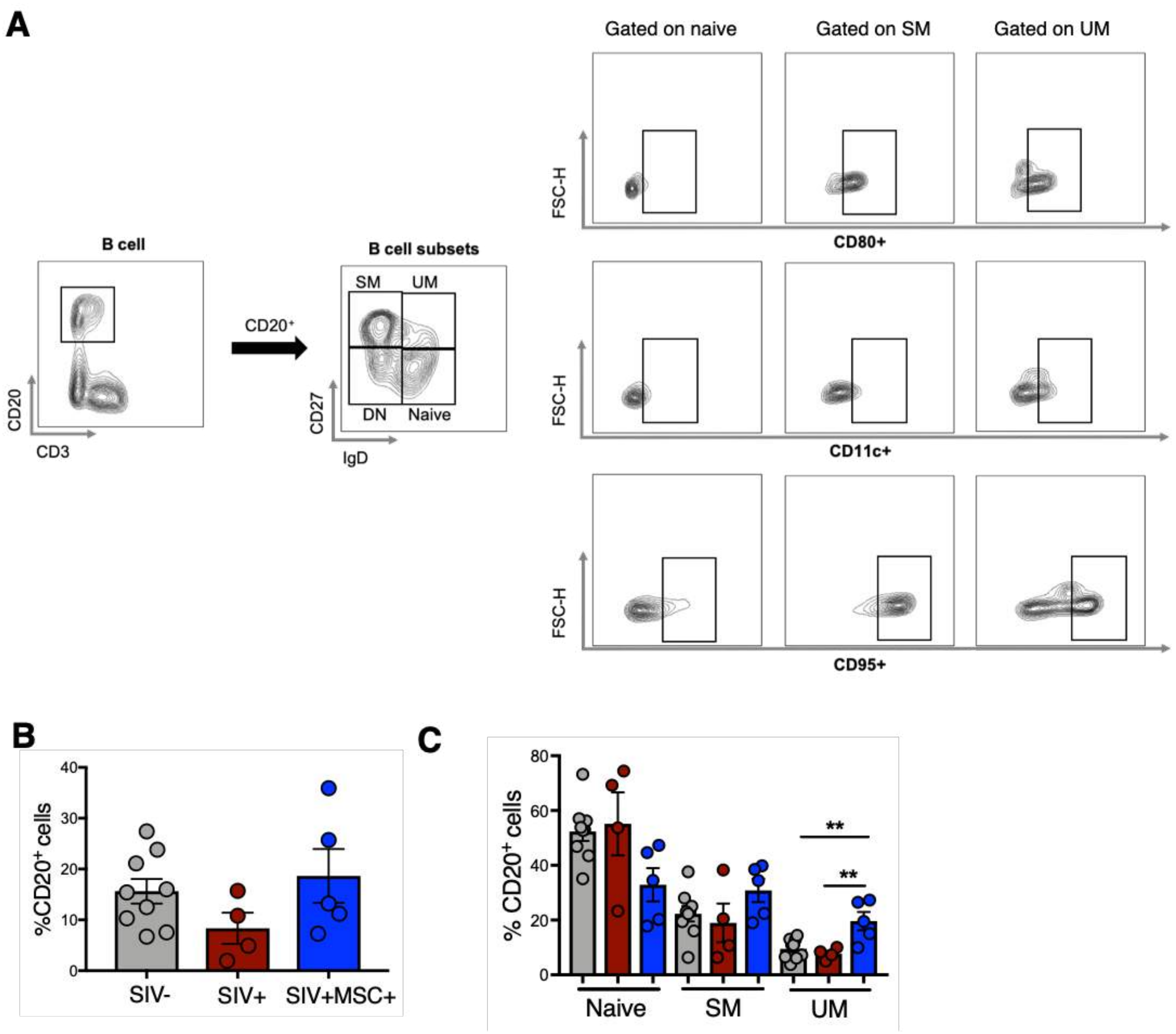

Supplementary Figure 4. Flow cytometric gating strategy and B cell subsets in peripheral blood. (A) Shown are percentages of B cells after gating on lymphocytes, singlets and live cells. B cells were identified by gating on CD3- CD20+ cells. Naïve (CD27- IgD+), switched memory (SM, CD27+ IgD-) and unswitched memory (UM, CD27+ IgD+) were identified by gating on CD27 and IgG. Cells were then gated on CD80, CD11c and CD95. **(B and C)** Frequency of total B cell (CD3- CD20+), naïve, switched and unswitched B cell populations (SIV-, n=9; SIV+, n =

4; SIV+MSC+, n = 5) at 70 dpi. Significance was determined using the 1-way ANOVA with Holm-Sidak post hoc testing for multiple comparisons (C). ** ($P \leq 0.01$).

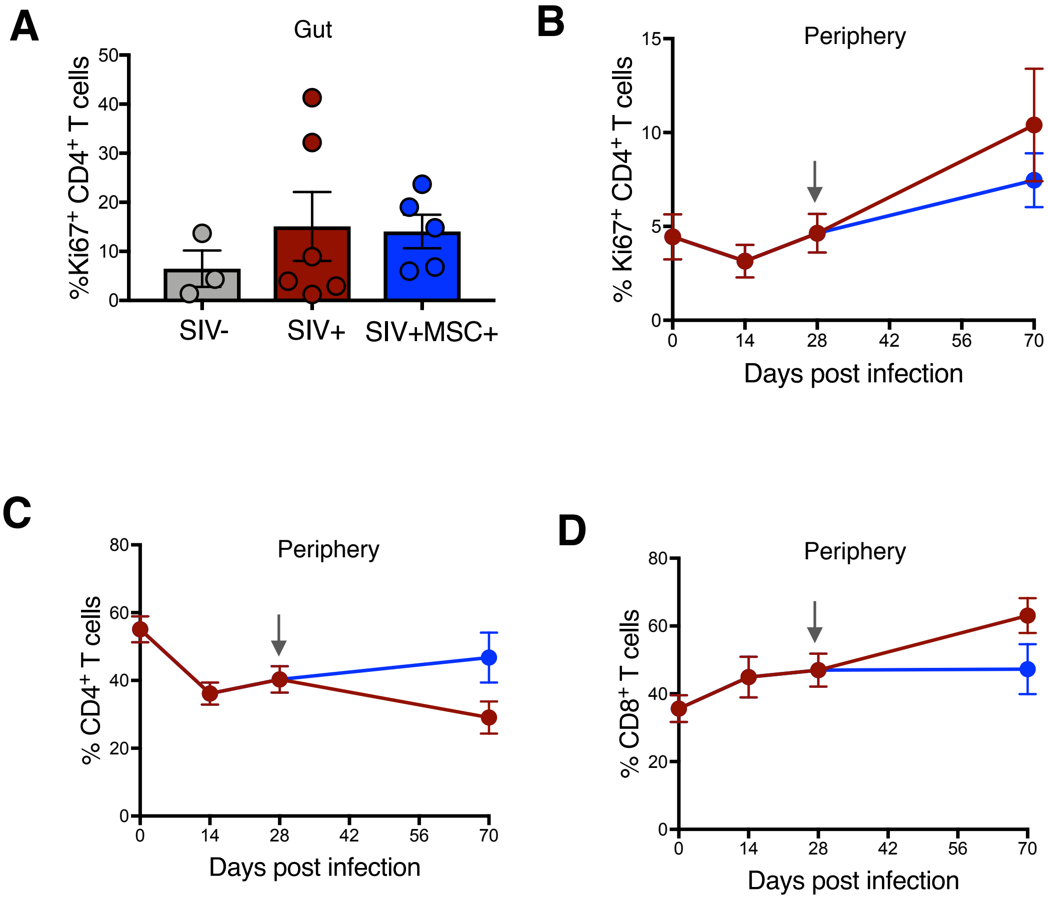

Supplementary Figure 5. Detection of T cells subsets. **(A)** Percentage of Ki67+ CD4+ T cells at 70 dpi in the colon (SIV- n=3, SIV+ n = 6, SIV+MSC+ n=5). **(B-D)** Longitudinal frequencies of Ki67+ CD4+ T cells, CD4+ T cells and CD8+ T cells in the periphery at 0, 14, 28 and 70 dpi gut (SIV+ n = 7 and SIV+MSC+ n = 5). Data represent the mean (± SEM) for each time point. Grey arrow represents the first MSC administration.

BIOCHEMICAL	log2fc SIV+/SIV-	p.value	q.value
equol sulfate	-5.217732482	0.002625418	0.141441655
lithocholate	-2.932070097	0.047654433	0.173975112
glutarate (C5-DC)	-2.706905966	0.048814512	0.173975112
heneicosapentaenoate (21:5n3)	-2.620059518	0.005975086	0.141441655
dodecanedioate (C12)	-2.617733563	0.012821685	0.141441655
N-acetyltryptophan	-2.23424581	0.020106576	0.163434898
cinnamoylglycine	-2.17719144	0.008159341	0.141441655
N-octanoylglycine	-2.073713503	0.017445605	0.16249916
docosahexaenoate (DHA; 22:6n3)	-2.008335692	0.011068407	0.141441655
10-undecenoate (11:1n1)	-1.951916855	0.00263701	0.141441655
valylglycine	-1.948219691	0.041745771	0.173928856
indoleacetylglutamine	-1.788619659	0.047937309	0.173975112
3-hydroxydecanoate	-1.739257929	0.043170587	0.173928856
eicosapentaenoate (EPA; 20:5n3)	-1.719526249	0.007003571	0.141441655
cis-4-decenoate (10:1n6)*	-1.712098214	0.010265927	0.141441655
2'-deoxyuridine	-1.693470996	0.006429544	0.141441655
docosapentaenoate (n6 DPA; 22:5n6)	-1.662201533	0.008180663	0.141441655
3-hydroxyoctanoate	-1.618034476	0.040539678	0.173928856
N-acetylaspartate (NAA)	-1.563719889	0.048814512	0.173975112
4-oxo-retinoic acid	-1.559311601	0.004841805	0.141441655
linolenate (18:3n3 or 3n6)	-1.550540301	0.012821685	0.141441655
3-bromo-5-chloro-2,6-dihydroxybenzoic acid*	-1.533123721	0.007016199	0.141441655
stearidonate (18:4n3)	-1.500334508	0.010979131	0.141441655
retinal	-1.500116264	0.019627115	0.16249916
3-hydroxymyristate	-1.494956865	0.033524	0.169683493
5-dodecenoate (12:1n7)	-1.490028363	0.025784235	0.16825866
arachidonate (20:4n6)	-1.474986858	0.007016199	0.141441655
dihomolinoleate (20:2n6)	-1.464341579	0.012821685	0.141441655
(16 or 17)-methylstearate (a19:0 or i19:0)	-1.448326748	0.017869873	0.16249916
lactobacillic acid	-1.426189881	0.012781783	0.141441655
3-hydroxystearate	-1.4040713	0.01683404	0.16249916
hexanoylglycine (C6)	-1.381922053	0.022471077	0.16825866
dihomolinolenate (20:3n3 or 3n6)	-1.343496804	0.012821685	0.141441655
myristoylcarnitine (C14)	-1.332193676	0.048814512	0.173975112
caprate (10:0)	-1.316021764	0.022501067	0.16825866
1-myristoyl-2-arachidonoyl-GPC (14:0/20:4)*	-1.295012842	0.008187777	0.141441655
1-linoleoyl-GPI* (18:2)*	-1.258525751	0.01964073	0.16249916
indoleacetate	-1.25568594	0.025784235	0.16825866
margarate (17:0)	-1.234022619	0.022531072	0.16825866
10-heptadecenoate (17:1n7)	-1.227315939	0.038088284	0.169683493
trans-2-hexenoylglycine	-1.215501918	0.023952962	0.16825866
2-palmitoyl-GPC* (16:0)*	-1.212316702	0.01964073	0.16249916
1-stearoyl-GPI (18:0)	-1.205428585	0.019586293	0.16249916
3,5-dichloro-2,6-dihydroxybenzoic acid	-1.175510371	0.025784235	0.16825866
1-linolenoyl-GPC (18:3)*	-1.169232287	0.033524	0.169683493
2-hydroxypalmitate	-1.156585948	0.011068407	0.141441655
3beta-hydroxy-5-cholestenoate	-1.150787074	0.038002593	0.169683493
stearate (18:0)	-1.146188899	0.011068407	0.141441655
undecenoylcarnitine (C11:1)	-1.140726018	0.038002593	0.169683493
2-hydroxynervonate*	-1.131843318	0.047937309	0.173975112
2-hydroxybehenate	-1.103638724	0.023561682	0.16825866
myristate (14:0)	-1.081039113	0.038088284	0.169683493
dodecadienoate (12:2)*	-1.069933394	0.033524	0.169683493
linoleate (18:2n6)	-1.069218441	0.029435937	0.169683493
octadecanedioate (C18)	-1.061882165	0.038088284	0.169683493
3-(3-hydroxyphenyl)propionate	-1.056419535	0.025718328	0.16825866
sphingomyelin (d17:1/14:0, d16:1/15:0)*	-1.033577356	0.029435937	0.169683493
1-palmitoleoyl-GPC* (16:1)*	-1.023341089	0.033524	0.169683493
oleate/vaccenate (18:1)	-1.023001894	0.025784235	0.16825866
2-hydroxystearate	-1.017932413	0.00368678	0.141441655
3-carboxy-4-methyl-5-propyl-2-furanpropanoate (CMPF)	-1.015852984	0.014816378	0.159644922
docosadienoate (22:2n6)	-1.01133176	0.033445231	0.169683493
adrenate (22:4n6)	-1.00847747	0.027540699	0.169683493
(14 or 15)-methylpalmitate (a17:0 or i17:0)	-1.007676789	0.038088284	0.169683493
3-hydroxyhexanoate	-0.93741855	0.029435937	0.169683493
hydroxy-CMPF*	-0.936166241	0.007016199	0.141441655
dihomo-linolenoyl-choline	-0.932100444	0.041970859	0.173928856
6-bromotryptophan	-0.930303198	0.02880637	0.169683493

palmitate (16:0)	-0.926001328	0.017079637	0.16249916
eicosanedioate (C20-DC)	-0.910346154	0.025784235	0.16825866
cysteinylglycine	-0.8863994	0.00511391	0.141441655
1-arachidonoyl-GPC* (20:4)*	-0.881376606	0.038088284	0.169683493
pentadecanoate (15:0)	-0.87683545	0.012821685	0.141441655
arachidate (20:0)	-0.868556834	0.038066857	0.169683493
cysteine sulfinic acid	-0.845540264	0.012811704	0.141441655
pyridoxate	-0.828002344	0.048814512	0.173975112
1-stearoyl-GPC (18:0)	-0.81368143	0.011068407	0.141441655
1-stearoyl-2-linoleoyl-GPI (18:0/18:2)	-0.796255449	0.007016199	0.141441655
sphingomyelin (d18:2/21:0, d16:2/23:0)*	-0.780291475	0.043077674	0.173928856
sphingomyelin (d18:2/14:0, d18:1/14:1)*	-0.777040495	0.038088284	0.169683493
1-(1-enyl-stearoyl)-GPE (P-18:0)*	-0.768343688	0.048814512	0.173975112
linoleoylcarnitine (C18:2)*	-0.755565257	0.048814512	0.173975112
3-methylcrotonylglycine	-0.754451132	0.043170587	0.173928856
cys-gly, oxidized	-0.738430708	0.038088284	0.169683493
1-(1-enyl-stearoyl)-2-arachidonoyl-GPE (P-18:0/20:4)*	-0.724604079	0.029435937	0.169683493
1-linoleoyl-2-arachidonoyl-GPC (18:2/20:4n6)*	-0.718814669	0.033524	0.169683493
retinol (Vitamin A)	-0.715690622	0.038088284	0.169683493
tryptophan	-0.695028261	0.012821685	0.141441655
threonate	-0.694885603	0.038088284	0.169683493
citrulline	-0.642531904	0.005997428	0.141441655
1-stearoyl-2-docosahexaenoyl-GPC (18:0/22:6)	-0.632366725	0.003690585	0.141441655
methylsuccinate	-0.61970496	0.00511391	0.141441655
5-methyluridine (ribothymidine)	-0.612962425	0.000751428	0.141441655
gamma-tocopherol/beta-tocopherol	-0.606169779	0.048814512	0.173975112
isocitrate	-0.586800664	0.043077674	0.173928856
aconitate [cis or trans]	-0.564952449	0.017079637	0.16249916
1-stearoyl-2-arachidonoyl-GPI (18:0/20:4)	-0.544940948	0.005997428	0.141441655
3-hydroxyindolin-2-one sulfate	0.546711948	0.032621784	0.169683493
1-carboxyethylphenylalanine	0.645307439	0.043170587	0.173928856
5,6-dihydrothymine	0.710683085	0.033524	0.169683493
3-ureidoisobutyrate	0.791542981	0.043147355	0.173928856
formiminoglutamate	0.821648995	0.033524	0.169683493
indolin-2-one	0.950843086	0.04856357	0.173975112
picolinate	1.00427424	0.011068407	0.141441655
N-acetylputrescine	1.004901066	0.017079637	0.16249916
3-ureidopropionate	1.058330318	0.011068407	0.141441655
N-acetyl-1-methylhistidine*	1.072934337	0.005997428	0.141441655
glycerol 3-phosphate	1.100730961	0.022531072	0.16825866
kynurenine	1.151278114	0.001563119	0.141441655
anthranilate	1.271902544	0.008046101	0.141441655
quinolinate	1.488138954	0.005997428	0.141441655
N-formylanthranilic acid	1.492824658	0.043170587	0.173928856
urate	1.664740426	0.008187777	0.141441655
3-methoxytyramine sulfate	3.508192085	0.026507093	0.169683493
glycochenodeoxycholate glucuronide (1)	4.783895949	0.045500264	0.173975112
2,3-dihydroxyisovalerate	4.865244142	0.030514659	0.169683493
carnosine	5.24654175	0.043170587	0.173928856

Supplementary Table 1. Significantly increased metabolites following SIV infection.

BIOCHEMICAL	log2fc SIV+MSC+/SIV+	p.value	q.value
carnosine	-7.481601691	0.028459737	0.074378169
3-methoxytyramine sulfate	-5.996513323	0.017365464	0.061799624
2,3-dihydroxyisovalerate	-5.490208715	0.049745991	0.103611059
4-acetamidobenzoate	-4.656082984	0.049745991	0.103611059
heme	-2.929626822	0.017622091	0.061799624
2-pyrrolidinone	-2.796731623	0.016604954	0.061799624
suberoylcarnitine (C8-DC)	-2.740783881	0.042680184	0.094350021
picolinate	-2.426197789	0.006169899	0.061183519
leucylhydroxyproline*	-2.012967128	0.034463821	0.089499495
2-hydroxyheptanoate*	-1.941549461	0.017622091	0.061799624
1-carboxyethylisoleucine	-1.767752299	0.006169899	0.061183519
indoleacetoylcarnitine*	-1.762487816	0.028459737	0.074378169
1-carboxyethylvaline	-1.753070354	0.006169899	0.061183519
N-acetyl-1-methylhistidine*	-1.751185545	0.017622091	0.061799624
octadecenedioylcarnitine (C18:1-DC)*	-1.740100966	0.028459737	0.074378169
tiglyl carnitine (C5)	-1.709954315	0.006169899	0.061183519
2S,3R-dihydroxybutyrate	-1.66735092	0.017622091	0.061799624
2-aminobutyrate	-1.660025582	0.006169899	0.061183519
N,N,N-trimethyl-alanylproline betaine (TMAP)	-1.631452879	0.006169899	0.061183519
alpha-hydroxyisovalerate	-1.626522448	0.006169899	0.061183519
propionylcarnitine (C3)	-1.617858436	0.010587137	0.061183519
N-acetyl-aspartyl-glutamate (NAAG)	-1.538713286	0.017622091	0.061799624
lanthionine	-1.520176575	0.042680184	0.094350021
alpha-hydroxyisocaproate	-1.495161632	0.006053705	0.061183519
2-hydroxy-3-methylvalerate	-1.444446422	0.006053705	0.061183519
N-acetylneuraminate	-1.430568227	0.027030077	0.074378169
gamma-glutamyl-2-aminobutyrate	-1.405759686	0.028459737	0.074378169
gamma-glutamylmethionine	-1.369458037	0.044609718	0.094350021
bilirubin	-1.265786576	0.006169899	0.061183519
alpha-ketobutyrate	-1.25817073	0.010411098	0.061183519
octadecanedioylcarnitine (C18-DC)*	-1.251747669	0.044609718	0.094350021
beta-hydroxyisovaleroylcarnitine	-1.239832209	0.028459737	0.074378169
biliverdin	-1.226789656	0.017622091	0.061799624
beta-alanine	-1.218418548	0.028459737	0.074378169
2-hydroxybutyrate/2-hydroxyisobutyrate	-1.213676571	0.028459737	0.074378169
arabitol/xylitol	-1.212374267	0.028459737	0.074378169
malonylcarnitine	-1.191391133	0.010587137	0.061183519
butyrylcarnitine (C4)	-1.169700297	0.028459737	0.074378169
2-methylbutyrylcarnitine (C5)	-1.159026908	0.010587137	0.061183519
N-acetylputrescine	-1.152255257	0.044609718	0.094350021
threonine	-1.061843235	0.006169899	0.061183519
isobutyrylcarnitine (C4)	-1.03549576	0.006169899	0.061183519
creatine	-1.026617677	0.006169899	0.061183519
deoxycarnitine	-1.015168155	0.017622091	0.061799624
alanine	-0.96189517	0.006169899	0.061183519
gamma-glutamylthreonine	-0.951338239	0.028459737	0.074378169
fructosyllysine	-0.947538844	0.028459737	0.074378169
hydroxyproline	-0.888460936	0.010587137	0.061183519
1-carboxyethylleucine	-0.881190596	0.044609718	0.094350021
hydroxy-N6,N6,N6-trimethyllysine*	-0.873878131	0.028459737	0.074378169
nonanoylcarnitine (C9)	-0.86470595	0.017622091	0.061799624
asparagine	-0.847075066	0.006169899	0.061183519
N6-carbamoylthreonyladenosine	-0.807296888	0.042680184	0.094350021
methionine	-0.774444641	0.006169899	0.061183519
N-acetylglucosamine/N-acetylgalactosamine	-0.764669543	0.006169899	0.061183519
gamma-glutamylvaline	-0.753371499	0.044609718	0.094350021
carnitine	-0.74704285	0.017622091	0.061799624
1,2-dipalmitoyl-GPC (16:0/16:0)	-0.669233733	0.028459737	0.074378169
gamma-glutamylhistidine	-0.660160058	0.028459737	0.074378169
3-hydroxyisobutyrate	-0.654025137	0.044609718	0.094350021
N-acetyl-isoputreanine	-0.647633587	0.017622091	0.061799624
valine	-0.646120684	0.044609718	0.094350021
gamma-glutamylphenylalanine	-0.634938628	0.017622091	0.061799624
gamma-glutamyltyrosine	-0.618567752	0.044609718	0.094350021
histidine	-0.584800649	0.017622091	0.061799624
ribitol	-0.570876034	0.017622091	0.061799624
1-palmitoyl-2-docosahexaenoyl-GPE (16:0/22:6)*	0.54620997	0.044609718	0.094350021
behenoyl sphingomyelin (d18:1/22:0)*	0.576476202	0.044609718	0.094350021
1-stearoyl-2-oleoyl-GPC (18:0/18:1)	0.593015043	0.044609718	0.094350021
1-stearoyl-2-linoleoyl-GPE (18:0/18:2)*	0.647039666	0.028459737	0.074378169

carotene diol (2)	0.657431522	0.044609718	0.094350021
methylsuccinate	0.660088925	0.017622091	0.061799624
4-hydroxyphenylpyruvate	0.668623387	0.044609718	0.094350021
lignoceroyl sphingomyelin (d18:1/24:0)	0.677633096	0.044609718	0.094350021
1-stearoyl-2-arachidonoyl-GPI (18:0/20:4)	0.68512921	0.006169899	0.061183519
1-arachidonoyl-GPE (20:4n6)*	0.690625367	0.044609718	0.094350021
glutamate	0.708462343	0.044609718	0.094350021
sphingomyelin (d18:1/14:0, d16:1/16:0)*	0.741322178	0.010587137	0.061183519
sphingomyelin (d18:2/14:0, d18:1/14:1)*	0.745475529	0.044609718	0.094350021
3,5-dichloro-2,6-dihydroxybenzoic acid	0.757449301	0.028459737	0.074378169
hydroxy-CMPF*	0.793120578	0.017622091	0.061799624
1-oleoyl-2-linoleoyl-GPE (18:1/18:2)*	0.842045939	0.028459737	0.074378169
glycerophosphorylcholine (GPC)	0.914730319	0.044609718	0.094350021
threonate	0.916751037	0.044609718	0.094350021
thyroxine	0.934942882	0.044609718	0.094350021
1-(1-enyl-stearoyl)-2-oleoyl-GPE (P-18:0/18:1)	0.95457994	0.017622091	0.061799624
1-oleoyl-2-docosahexaenoyl-GPC (18:1/22:6)*	0.987259558	0.010587137	0.061183519
1-(1-enyl-stearoyl)-2-linoleoyl-GPE (P-18:0/18:2)*	0.987837003	0.017622091	0.061799624
1-palmitoyl-GPC (16:0)	0.992351032	0.028459737	0.074378169
1-(1-enyl-palmitoyl)-GPE (P-16:0)*	1.010818047	0.017622091	0.061799624
3-methylcrotonylglycine	1.012915561	0.017622091	0.061799624
butyrylglycine (C4)	1.059320736	0.017622091	0.061799624
1-arachidonoyl-GPC* (20:4)*	1.062663696	0.010587137	0.061183519
homoarginine	1.118585989	0.006169899	0.061183519
1-stearoyl-GPE (18:0)	1.134704936	0.028459737	0.074378169
3-bromo-5-chloro-2,6-dihydroxybenzoic acid*	1.160359117	0.010587137	0.061183519
cysteine sulfinic acid	1.169877918	0.028100064	0.074378169
1-linoleoyl-2-arachidonoyl-GPC (18:2/20:4n6)*	1.199266181	0.006169899	0.061183519
1-linoleoyl-GPC (18:2)	1.228041289	0.028459737	0.074378169
2-hydroxypalmitate	1.258430985	0.044609718	0.094350021
1-(1-enyl-palmitoyl)-GPC (P-16:0)*	1.272501259	0.028459737	0.074378169
1-oleoyl-GPC (18:1)	1.286746681	0.028459737	0.074378169
1-myristoyl-2-arachidonoyl-GPC (14:0/20:4)*	1.295683523	0.028459737	0.074378169
1-palmitoleoyl-2-linolenoyl-GPC (16:1/18:3)*	1.335383433	0.017622091	0.061799624
2-hydroxystearate	1.341706284	0.010411098	0.061183519
1-stearoyl-2-linoleoyl-GPI (18:0/18:2)	1.357494429	0.006169899	0.061183519
1,2-dilinoleoyl-GPC (18:2/18:2)	1.39196874	0.006169899	0.061183519
phenylacetylglutamine	1.401389153	0.044609718	0.094350021
10-undecenoate (11:1n1)	1.405502892	0.017622091	0.061799624
1-palmitoyl-2-arachidonoyl-GPI (16:0/20:4)*	1.414674318	0.010587137	0.061183519
retinol (Vitamin A)	1.416256615	0.010587137	0.061183519
3-hydroxyhippurate	1.427874504	0.022043575	0.074378169
1-oleoyl-2-docosahexaenoyl-GPE (18:1/22:6)*	1.432828545	0.017622091	0.061799624
phenylacetylglycine	1.44168396	0.028459737	0.074378169
3beta-hydroxy-5-cholestenoate	1.442677825	0.04412557	0.094350021
leucylalanine	1.454436088	0.017365464	0.061799624
1-stearoyl-GPC (18:0)	1.464477836	0.006169899	0.061183519
oleoyl-linoleoyl-glycerol (18:1/18:2) [2]	1.493128794	0.006169899	0.061183519
6-bromotryptophan	1.494618883	0.028100064	0.074378169
palmitoyl-linoleoyl-glycerol (16:0/18:2) [2]*	1.499613849	0.010587137	0.061183519
1-(1-enyl-stearoyl)-GPE (P-18:0)*	1.503862029	0.017622091	0.061799624
1-palmitoyl-2-linoleoyl-GPI (16:0/18:2)	1.510887005	0.006169899	0.061183519
1-palmitoyl-2-oleoyl-GPI (16:0/18:1)*	1.531050876	0.010587137	0.061183519
arachidonate (20:4n6)	1.539182232	0.017622091	0.061799624
1-oleoyl-2-arachidonoyl-GPI (18:1/20:4)*	1.540342259	0.010587137	0.061183519
docosapentaenoate (n6 DPA; 22:5n6)	1.564910216	0.010411098	0.061183519
picolinoylglycine	1.570293955	0.024932456	0.074378169
p-cresol glucuronide*	1.591896431	0.028100064	0.074378169
oleoyl-linoleoyl-glycerol (18:1/18:2) [1]	1.59232199	0.006169899	0.061183519
p-cresol sulfate	1.596099303	0.010587137	0.061183519
dihomolinolenate (20:3n3 or 3n6)	1.640998438	0.017622091	0.061799624
3-hydroxystearate	1.641611476	0.006053705	0.061183519
3-indoxyl sulfate	1.663004514	0.010587137	0.061183519
palmitoyl-linoleoyl-glycerol (16:0/18:2) [1]*	1.67363603	0.010587137	0.061183519
6-hydroxyindole sulfate	1.674419988	0.017622091	0.061799624
4-oxo-retinoic acid	1.677501427	0.035174861	0.090204178
2-stearoyl-GPE (18:0)*	1.730596619	0.017365464	0.061799624
2-palmitoyl-GPC* (16:0)*	1.763434508	0.010587137	0.061183519
retinal	1.782327675	0.010411098	0.061183519
linoleoyl-linoleoyl-glycerol (18:2/18:2) [1]*	1.789837853	0.006169899	0.061183519
1-linoleoyl-GPA (18:2)*	1.793632323	0.006169899	0.061183519
palmitoyl-oleoyl-glycerol (16:0/18:1) [2]*	1.853941265	0.017622091	0.061799624
1-ribosyl-imidazoleacetate*	1.883247668	0.028459737	0.074378169
N-methylproline	1.930450673	0.017622091	0.061799624
linoleoyl-linolenoyl-glycerol (18:2/18:3) [1]*	1.941226564	0.028459737	0.074378169
1-linoleoyl-2-linolenoyl-GPC (18:2/18:3)*	1.955987792	0.006169899	0.061183519

1-stearoyl-2-oleoyl-GPI (18:0/18:1)*	1.974733913	0.010587137	0.061183519
docosahexaenoate (DHA; 22:6n3)	2.005016062	0.010587137	0.061183519
1-linoleoyl-GPI* (18:2)*	2.027711681	0.010587137	0.061183519
1-linolenoyl-GPC (18:3)*	2.032388012	0.010587137	0.061183519
palmitoyl-oleoyl-glycerol (16:0/18:1) [1]*	2.033937001	0.010587137	0.061183519
oleoyl-oleoyl-glycerol (18:1/18:1) [1]*	2.047781907	0.006169899	0.061183519
1-palmitoleoyl-GPC* (16:1)*	2.069231452	0.010587137	0.061183519
1-stearoyl-GPI (18:0)	2.246094543	0.006169899	0.061183519
oleoyl-oleoyl-glycerol (18:1/18:1) [2]*	2.275772766	0.006169899	0.061183519
palmitoylcholine	2.323572496	0.044609718	0.094350021
1-lignoceroyl-GPC (24:0)	2.332098181	0.028100064	0.074378169
4-allylphenol sulfate	2.339887309	0.027030077	0.074378169
palmitoleoyl-linoleoyl-glycerol (16:1/18:2) [1]*	2.443806894	0.010587137	0.061183519
3-phenylpropionate (hydrocinnamate)	2.464139538	0.04412557	0.094350021
diacylglycerol (16:1/18:2 [2], 16:0/18:3 [1])*	2.498572284	0.010587137	0.061183519
linoleoyl-linolenoyl-glycerol (18:2/18:3) [2]*	2.578432302	0.00734473	0.061183519
heneicosapentaenoate (21:5n3)	2.624217659	0.028100064	0.074378169
1-oleoyl-GPI (18:1)	2.812955637	0.017365464	0.061799624
hippurate	2.91708645	0.028459737	0.074378169
palmitoleoyl-arachidonoyl-glycerol (16:1/20:4) [2]*	3.004934795	0.035174861	0.090204178
1-palmitoyl-GPI* (16:0)	3.03685194	0.010411098	0.061183519
cinnamoylglycine	3.237277871	0.017365464	0.061799624
docosahexaenoylcholine	3.274370086	0.017365464	0.061799624
equol glucuronide	3.484777815	0.011163994	0.061799624
N-acetyltryptophan	3.61102606	0.015127246	0.061799624
deoxycholate	3.679342924	0.017622091	0.061799624
4-methylcatechol sulfate	3.767300958	0.04412557	0.094350021
lithocholate	4.032029996	0.011163994	0.061799624
dihomo-linolenoyl-choline	4.160179239	0.036571191	0.093202483
indolepropionate	4.597916618	0.017622091	0.061799624
4-ethylphenyl sulfate	5.578175588	0.010411098	0.061183519
equol sulfate	5.876817264	0.016604954	0.061799624

Supplementary Table 2. Significantly increased metabolites following MSC administration.

Study Group	Animals/group	Weight (kg)	Sex (F:M)	Age (yrs.)
SIV-	18	9.6 ± 2.3	2:16	6.0 ± 1.9
SIV+	12	9.7 ± 2.8	3:9	8.0 ± 1.1
SIV+MSC+	5	7.2 ± 1.3	5:0	6.8 ± 1.9

Supplementary Table 3. Characteristics of animals in the study groups

Antigen/Reagent	Conjugate	Clone	Company	Catalog Number
CD3	Pacific Blue	SP34-2	BD	558124
CD3	AF700	SP-234	BD	557917
CD4	PerPCy5.5	OKT4	Biolegend	317427
CD4	BV650	L200	BD	563737
CD8	PE Dazzle 594	RPA-T8	Biolegend	301057
CD8	PerCPCy5.5	SK1	BD	341049
CD8	BV510	SK1	BD	563919
CD45	AF700	D058-1283	BD	561288
Ki67	AF488	B56	Fisher Scientific	BDB561165
CD95	APC	DX2	BD	558814
CD95	BUV737	DX2	BD	564710
CXCR5	PE	MU5UBEE	Invitrogen	12-9185-411G1
CXCR5	BV421	MU5BEE	Invitrogen	48-9185-4
PD-1	PECyanine7	EH12.2H7	Biolegend	329918
Granzyme B	PECF594	GB11	BD	562462
CD20	BV421	2H7	Biolegend	302328
CD20	APC-H7	L27	BD	641396
CD11c	BV650	S-HCL-3	BD	744437
CD21	PECyanine7	B-ly4	BD	561374
CD27	Pacific Blue	M-T271	Biolegend	356413
CD80	PE	L307-4	BD	557227
IgD	FITC		Southern Biotech	2030-02
IFNγ	PECyanine7	B27	BD	557643
TNF-α	AF488	Mab11	Invitrogen	53-734971
Live-dead marker	APCCy7	NA	Invitrogen	L34976A
Live-dead marker	Aqua	NA	Invitrogen	L34957

Supplementary Table 4. Flow cytometry reagents.
